# A comparative analysis of machine learning, deep learning, and ensemble learning models for suicidal ideation detection on social media

**DOI:** 10.1371/journal.pmen.0000721

**Published:** 2026-10-22

**Authors:** Andualem Woldegiorgis, Mohammed Abebe, Durga Prasad Sharma, Worku Jimma

**Affiliations:** 1 Faculty of Computing and Software Engineering, Arba Minch University, Arba Minch, Ethiopia; 2 College of Engineering and Technology, Bonga University, Bonga, Ethiopia; 3 Expat Professor, Arba Minch University, Arba Minch, Ethiopia; 4 Faculty of Computing and Informatics, Jimma University, Jimma, Ethiopia; Instituto Federal do Maranhão: Instituto Federal de Educacao Ciencia e Tecnologia do Maranhão, BRAZIL

## Abstract

Suicidal ideation is the consideration of self-inflicted death and has emerged as a serious and rising concern for worldwide public health systems, contributing to over 800,000 deaths annually worldwide. Individuals express intentions, distress, and thoughts of self-harm and death, thereby offering a crucial and unprecedented avenue for timely psychological intervention and surveillance. The core problem addressed by this research is the pronounced global insufficiency in detecting and mitigating suicidal ideation across linguistically diverse environments, specifically focusing on low-resource languages. Previous computational studies have primarily focused on English and other widely used languages, leaving a critical research gap for contexts such as Amharic. The main aim of this study was to investigate and to develop a robust and optimal deep learning model for identifying suicidal ideation in Amharic social media content. A total of 5,428 postings from social media such as Facebook and Telegram were carefully collected, manually annotated, and processed to provide the first publicly available dataset on Amharic suicidal ideation. After preprocessing, Word2Vec, GloVe, and FastText were among the word embedding techniques used for feature engineering. Five deep learning models and four machine learning models were then compared in this study. Based on the experimental results, ensemble learning models achieved slightly higher performance than the individual deep learning models in suicidal ideation detection. Specifically, hard voting ensemble learning models achieved the highest accuracy (83.3%) and precision (86.55%). CNN-BiLSTM achieved an accuracy of 81.45%, while the BiLSTM model achieved an accuracy of 81.4%, indicating that bidirectional sequence learning effectively captures contextual information in Amharic texts. Beyond its strong empirical performance, this study contributes a robust detection framework and introduces a public Amharic dataset, thereby establishing a foundational methodological resource for mental health research. Future studies should focus on developing scalable datasets and context-aware deep learning models that effectively handle multimodal data, multilingual transformer models, fusion-based features, and addressing bias in low-resource computational linguistics.

## 1 Introduction

Suicide is defined as intentional self-harm done to end one's own life. Suicidal ideation, which is the act of contemplating self-inflicted death, as well as the creation of a suicide plan and potentially lethal or nonfatal suicide attempts, sometimes precedes it [1,2]. Suicide-related deaths have become the most serious public health issue. According to the World Health Organization, more than 800,000 people worldwide die by suicide each year, equivalent to one death every 40 seconds [3]. It was the third most common cause of death worldwide in 2021, highlighting the critical need for comprehensive mental health care and increased awareness to successfully address this growing public health epidemic [4]. Suicide rates in Ethiopia increased gradually between 2006 and 2023 [2,4].

Promoting digital literacy, increasing awareness of mental health concerns, and putting early interventions into place to encourage support and lessen stigma are all necessary to combat this global health issue [5,6]. Staying alone, having little money, having little social support, experiencing psychological discomfort, and being addicted to the internet are some of the characteristics linked to an increased risk of suicidal ideation [7].

Social media platforms have transformed traditional media by enabling rapid communication, sharing of opinions, and information exchange. These platforms are increasingly used, particularly by younger generations, to discuss personal struggles, including suicidal thoughts and self-harm [8,9].

Monitoring and analyzing social media for signs of suicidal ideation is crucial for enabling prompt and effective interventions. Such strategies can help create safer online environments by preventing suicide, reducing self-harming behaviors, supporting at-risk individuals, and limiting the spread of suicide-related content. Numerous studies have explored the use of machine learning models for detecting suicidal ideation in social media content, with deep learning (DL) models emerging as a prevalent trend in the field [9–11].

Prolonged use of social networking platforms has been associated with increased levels of stress, anxiety, and depression, which can contribute to feelings of loneliness and inadequacy [8,12].

Rising rates of suicidal thoughts are largely caused by these emotional and psychological difficulties, especially in vulnerable populations like young adults between the ages of 15 and 29 [13]. The majority of research on suicidal ideation detection has been on commonly used languages, such as English, while resource-constrained languages have been given less attention in research [14,15].

Although numerous machine learning, deep learning, and ensemble learning models have demonstrated promising performance for suicidal ideation detection, most have been developed using high-resource languages such as English and benchmark datasets from Reddit and Twitter (X). This model cannot be directly generalized to Amharic because of its unique script, complex morphology, orthographic variation, limited linguistic resources, and scarcity of publicly available annotated datasets. Moreover, linguistic and cultural differences influence how suicidal thoughts are expressed, making language-specific models essential for reliable detection. In addition, there has been limited research effort in Ethiopia investigating suicide attempts using artificial intelligence (AI), machine learning (ML), and deep learning approaches in the context of social media [16]. Therefore, this study addresses these gaps by developing the first publicly available Amharic suicidal ideation dataset and conducting a comprehensive comparison of machine learning, deep learning, and ensemble learning models to establish a benchmark for suicidal ideation detection for low-resource languages and to evaluate its performance using standard metrics [17]. Furthermore, this study provides a benchmark framework for future research work that will extend the benchmark by incorporating emojis, multimodal, multilingual transformer architectures such as mBERT, XLM-R, AfriBERTa, AfroLM, and other language models, to investigate whether the contextual multilingual representations further improve suicidal ideation detection in low-resource languages. Based on these objectives, this study aims to address the following research questions.

**Research Questions:**

**RQ1:** How can a benchmark dataset for Amharic suicidal ideation detection be developed from social media?
**RQ2:** What are the most effective deep learning models that demonstrate the highest performance in detecting nuanced indicators of suicidal ideation on social media?
**RQ3:** What are the contextual research gaps towards the future research perspective for linguistic localization of scalable social media datasets, and context-aware deep learning for handling multimodal and multilingual classification tasks?

## 2 Related works

A study [13], proposed an automated system for identifying and detecting suicidal ideation on social media through deep learning and machine learning models. The study uses Tf-IDF and Word2Vec embeddings on a Reddit dataset of 232,000 posts. The CNN-BiLSTM achieved 95% accuracy, exceeding XGBoost’s 91.5%. The experimental result presents the effectiveness of deep learning, particularly CNN-BiLSTM, for early detection and prevention of suicidal behavior online.

A study in [17], applied several machine learning models to detect suicidal ideation, including Random Forest (RF), Naive Bayes (NB), gradient-boosted classification tree (GBDT), and XGBoost. The experimental results demonstrated that the Random Forest (RF) model achieved optimal performance. Using 5-fold cross-validation, it attained an accuracy of 98.92% and an F1-score of 98.92%, outperforming the other evaluated models.

A similar study in [18], presented a comprehensive machine learning model for the automatic detection of suicidal ideation on social media posts. The study employed four word embedding approaches, such as Word2Vec, GloVe, MPNet, and GPT-3 in combination with logistic regression, decision trees, support vector machines, multilayer perceptron, and random forest. The experimental results demonstrated that the multilayer perceptron model with GPT-3 embedding achieved the best performance, attaining accuracy, recall, and F1-score values ranging from 83 to 91%.

The same study in [14], explores different deep learning models for suicidal ideation detection using a combination with explainable artificial intelligence to interpret the model’s decision. Another study [12], investigates the application of transformer- based models like BERT and deep neural networks with word embedding to identify suicidal thoughts and depression in social media discourse. It received F1-scores of 0.97, 0.75, and 0.68, respectively, when tested on the Suicide Detection, CEASEv2.0, and SWMH datasets. The study noted challenges with data imbalance, loud language, and ethical concerns like prejudice and privacy.

A study [6], investigates the critical issue of suicide by analyzing emotions in suicide-related texts drawn from social media and suicide notes. It introduces a novel context dynamic masking based on bidirectional long short-term memory along with a multi-head self-attention and convolutional neural network (CoDyn-BMHSA-CNN) model for emotion detection, achieving an accuracy of 98.2% on the Reddit dataset and 97.4% on the CEASE-v2.0 dataset. In addition, the model recorded a precision score of 96.8% and 94.1%, along with recall values of 95.2% and 92.7%, respectively.

The same study in [5,13] proposed a CNN-BiLSTM deep learning approach and an LLM, i.e., GPT, on the same dataset, achieving performance over classical machine learning NB, SVM, and deep learning algorithms, i.e., RN, ACL, CNN-LSTM, Triple embedded C-BiLSTM, and LLMs, i.e., BERT, RoBERTa, and GPT2. The experimental results show that C-BiLSTM and GPT models outperformed baseline deep learning and LLM baseline models, achieving an accuracy of 94.5% and 97.69%, respectively.

A study in [19] proposes a novel deep learning approach that converts text and emojis from social media posts into 2D images for suicide ideation detection. Using pre-trained CNN models (VGG-16, ResNet-50, AlexNet) on the Reddit dataset, the method achieved 99.99% accuracy, outperforming traditional machine learning models. The same study [20], presents an ensemble deep learning model that integrates an LSTM-Attention-CNN model to detect suicidal ideation on social media posts applied on the Reddit dataset. The model classifies posts into four risk levels (No, Low, Moderate, Severe). The CNN model extracts important textual properties, the LSTM model captures contextual dependencies, and the attention mechanism draws attention to emotional words. The proposed model demonstrated superior performance compared to the baseline learning model, with an accuracy of 90.3% and an F1-score of 92.6%.

A study [21], developed a deep learning model that combines BERT and BiLSTM to create a unique model for identifying depression in social media text. The model achieved an accuracy of 88.95%, precision of 85.66%, recall of 93.81%, and F1 score of 89.55%.

These results demonstrated that the suggested BERT-BiLSTM model, which was combined with a knowledge graph, surpassed conventional techniques in identifying depression-related emotions. Another study [22], combines Grey Wolf Optimization with Recursive Feature Elimination and Stepwise Feature Selection to investigate deep learning models and present a unique feature selection technique for identifying suicidal ideation on social media. The results show an improvement in performance in detection while having lower dimensionality, achieving an accuracy of 97% and 89% on Twitter and Reddit, respectively.

A study [23], developed a hybrid model that combines transformer models with linguistic and metadata features to refine depression detection. By applying Easy Data Augmentation (EDA), the authors observed a substantial performance increase, raising the F1-score from 72.59% to 84.15%. The final model achieved balanced scores across precision, recall, and F1 (averaging ~84%). Beyond performance, the research highlights successful strategies for managing imbalanced datasets and demonstrates cross-platform adaptability.

According to the researcher in [24], a novel framework was developed that combines word embedding fusion, deep learning, generative adversarial networks, and evolutionary algorithms to classify suicidal ideation postings from Twitter. The result shows that the CNN model with FastText feature embedding achieved a 98% F1-score for high risk and 99% for low risk. With the addition of Genetic Algorithms and Generative Adversarial Networks (GANs), the LSTM + Fusion model had an accuracy of 89.73%. They concluded that using deep learning in conjunction with several feature extraction approaches resulted in a reliable predictive model for determining the probability of suicide in certain tweets.

A study in [8], examines the efficiency of numerous algorithms on diverse datasets and investigates machine learning models for identifying suicidal ideation on social media posts.

Another study [25], introduces a machine learning model for identifying suicidal risk levels in social media posts using the Enhanced Feature Engineering Approach for Suicidal Risk Identification (EFASRI). Researchers looked at Reddit and Twitter posts and categorized them into high, moderate, and no-risk groups with guidance from mental health professionals. With an accuracy of 96.33%, EFASRI employs a range of linguistic and statistical factors, including TF-IDF, LSI, ARS, and topic modeling, to identify indicators of distress. When combined with EFASRI, the XGBoost algorithm performed better than traditional methods. This approach offers a potentially helpful tool for early suicidal thought detection and timely mental health intervention.

A similar study in [26]], developed an AI-based predictive model that used machine learning and natural language processing to identify suicide ideation in Twitter data with 83% recall, 88% precision, and 85% accuracy. A precision-recall AUC score of 93% showed that the model performed well in detecting high-risk patients with few false positives.

As a result, an analysis of recent research works demonstrates a significant effort to identify suicidal ideation on social media through the use of deep learning and machine learning models [27]. Numerous methods have been used, such as CNN-BiLSTM [28], CNN-LSTM [29], transformer architectures (e.g., BERT, GPT) [30], and hybrid models that combine GANs, genetic algorithms, and optimization techniques (e.g., Grey Wolf Optimization), with high accuracy ranging from 88% to 99%. The majority of research, however, concentrates on unimodal textual data, which is frequently restricted to certain platforms like Reddit or Twitter. This presents several difficulties, such as data imbalance, a lack of cross-platform validation, and minimal integration of multimodal characteristics (such as images, memes, video, audio, or emojis). Furthermore, the majority of studies on suicidal ideation have mostly concentrated on widely spoken languages, especially English [31]. On the other hand, languages with low resources have received low attention, and there aren't many publicly accessible datasets in these languages. This gap restricts the creation of effective detection models and the adaptability of existing techniques to different linguistic and cultural contexts.

Recently, pre-trained multilingual transformer models have become a dominant approach for text classification in low-resource natural language processing. Unlike conventional deep learning models that rely on static word embeddings such as Word2Vec, GloVe, and FastText, transformer-based architectures generate contextualized representations that capture semantic and syntactic dependencies more effectively. A BERT model introduced contextual language modeling and significantly outperformed previous recurrent neural networks across benchmark tasks [32]. Subsequently, multilingual BERT (mBERT) extends its capacity to more than 100 languages, demonstrating strong Zero-shot cross-lingual transfer for multilingual text classification tasks [33]. Although multilingual transformers have shown remarkable success, African languages remain underrepresented during pretraining. A study in [34], proposed multilingual adaptive fine-tuning (MAFT) models for 17 African languages. Their experiment demonstrates competitive performance with low computational requirements. Similarly, a study in [31], introduced AfroLM, a multilingual pretrained language model for 23 African languages. The proposed AfroLM demonstrated the importance of language-specific pretraining for African languages by outperforming mBERT, XLM-R, and AfriBERTa models on several NLP tasks, including text classification and sentiment analysis, despite being trained on a significantly smaller corpus than current multilingual models.

Despite these advances, very limited research has investigated transformer-based approaches for Amharic suicidal ideation detection. Most recent studies continue to focus on high-resource languages such as English-language Reddit and Twitter datasets, while publicly available benchmark datasets for Amharic mental health applications remain scarce. Consequently, this study focused on developing a benchmark Amharic suicidal ideation dataset and systematically comparing machine learning, deep learning, and ensemble learning models. Future work will extend this benchmark by incorporating emojis, multimodal, multilingual transformer architectures, such as mBERT, XLM-R, AfriBERTa, AfroLM, and other large language models, to investigate whether contextual multilingual representations further improve suicidal ideation detection in low-resource languages.

## 3 Materials and methods

This study focuses on Amharic social media content and uses a data-driven approach to identify suicidal ideation in low-resource languages. To establish the research context and identify existing knowledge gaps, a review of the literature was conducted. Relevant scholarly publications published between 2020 and 2025 that were indexed in reputable databases like IEEE Xplore, Scopus, Springer, the ACM Digital Library, and Google Scholar were compiled in this review.

The terms ‘machine learning’, ‘deep learning’, ’suicidal ideation detection’, ’depression detection’, and ‘mental health’ are the most important. The study develops the first publicly available dataset of Amharic suicidal ideation from Facebook and Telegram using preprocessing methods such as tokenization, stop-word removal, and normalization. Then the dataset was divided into test and training sets. The machine learning models, i.e., LR, RF, SVM, and Naïve Bayes, and deep learning models such as CNN, CNN-BiLSTM, LSTM, and CNN-LSTM, were among the deep learning models that were employed. Accuracy, precision, recall, and F1-score were among the metrics used to analyze and evaluate their performance. The following sequential steps comprise the suggested architectural framework, which is depicted in Fig 1: data collection, data preprocessing, feature engineering, model selection, parameter adjustment, and outcome analysis.

**Fig 1:**
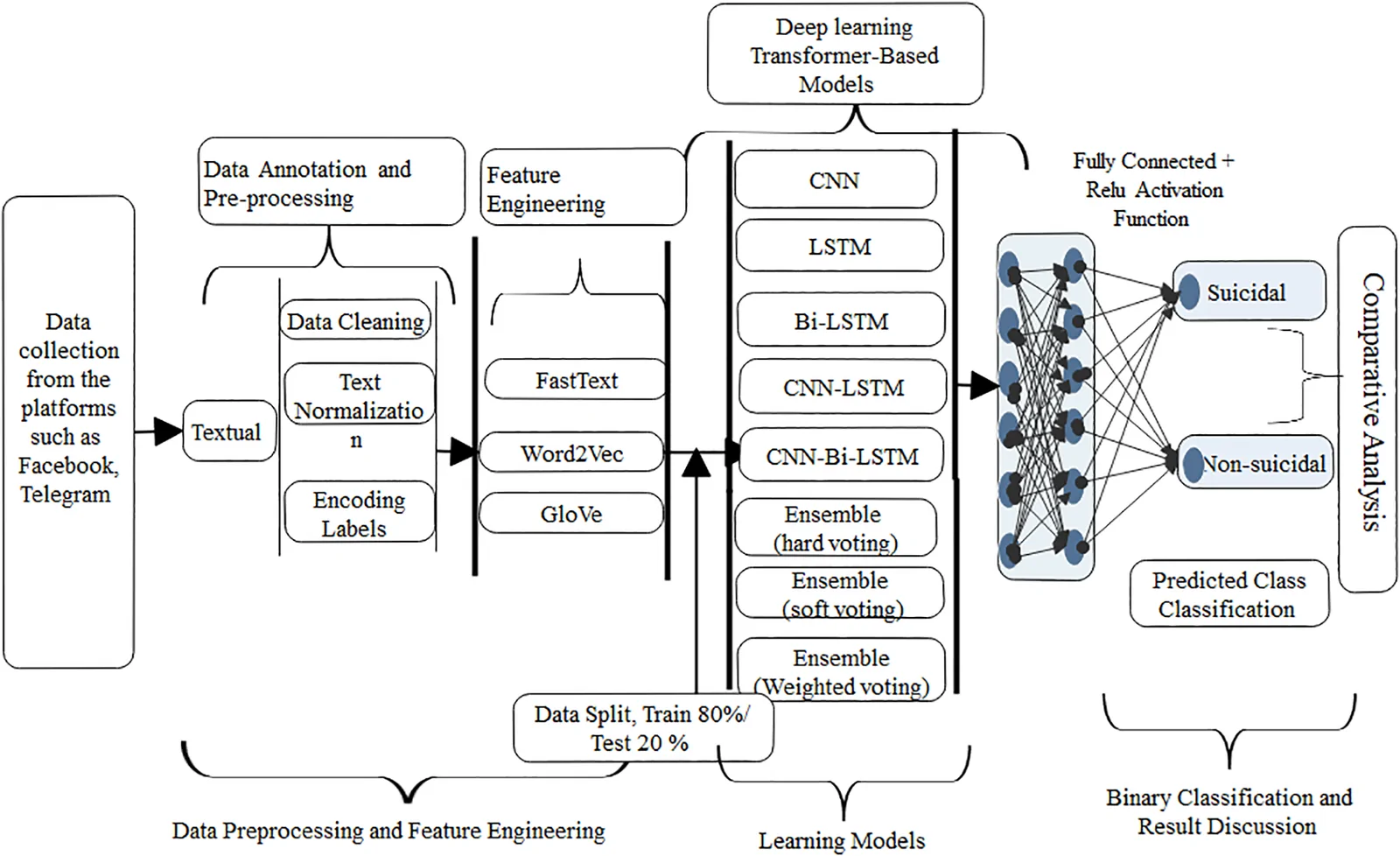
Proposed architectural framework.

As illustrated in Fig 1, the textual information was gathered from Telegram and Facebook. Then, word embedding techniques like FastText [35], GloVe [36], and Word2Vec [20,37] were used to preprocess and feature engineer the data. In this study, all embeddings were trained from scratch using collected Amharic data to ensure domain relevance and linguistic consistency. Several machine learning, deep learning, and transformer-based models were then used with the obtained features. Due to the small and imbalanced nature of the dataset, we used a stratified split strategy and a stratified 10-fold cross-validation, which preserves the original class distribution. Due to the small size of the dataset, an 80/20% split was used to separate the dataset into training and test sets [38]. The chosen models then carried out binary classification, which was assessed using conventional performance criteria [39].

### 3.1 Data collection and preprocessing

In this study, the data were collected exclusively from publicly accessible Facebook and Telegram posts. The exact data collection period was not systematically recorded during the initial data-gathering process, and the data collected were selected based on predefined annotation guidelines. To protect the privacy of individuals in the dataset, all personally identifiable information, including direct quotes, usernames, profile information, and URLs, has been removed and the dataset was used solely for academic and research purposes.

Platform and conditions compliance: The collection and analysis of the dataset were conducted using publicly available content from Facebook and Telegram. The study did not access private accounts, private groups, direct messages, or other restricted content, and no access control privacy mechanisms were circumvented. The collection and subsequent analysis were conducted in accordance with applicable terms, policies, and conditions governing access to publicly available content on respective platforms [40].

A domain expert labeled the data using a broad annotation standard that had been established [41,42]. A domain expert used broad suicidal keywords to annotate the dataset, which is divided into two classes: suicidal and non-suicidal. Table 1, shows a total of 5,428 Amharic social media posts that were classified as suicidal, labeled as 1, when they contained explicit or implicit indications of suicidal thoughts, intentions, plans, self-harm ideation, hopelessness related to ending one’s life, or expressions indicating a desire to die, with a total of 1,987 data points, and non-suicidal, labeled as 0, that did not contain suicidal intent or life-ending expressions, with a total of 3441 using a label data. A total of 985 commonly occurring Amharic stop words were collected, and 111 distinct terms and phrases were used as suicidal word indicators. To reduce noise and to ensure consistency, non-linguistic content such as URLs, emojis, special symbols, Ethiopic numbers, and stop words was removed. The repeated punctuation and characters were reduced. In addition, text was tokenized and normalized to ensure consistent representation of the linguistic unit.

**Table 1:** Inter-rater agreement.

	Agreed instance	Disagreed instance	Total annotated	Observed agreement (%)	Cohen's Kappa agreement
Suicidal	1913	70	1987	96.28	
Non-suicidal	3334	108	3441	96.89	
**Overall**	**5247**	**178**	**5428**	**96.72**	**0.93**

Fig 3, illustrates the dataset distribution used in this research study. This two-class dataset offers a basis for precisely identifying suicidal thoughts in Amharic social media. Because the same phoneme is represented by several Amharic letters, the Amharic language has considerable orthographic variety. Character-level normalization was used, as shown in Fig 2, to combine characters that are semantically and visually identical. Characters with similar punctuation are mapped into a single canonical form, which significantly reduces vocabulary size and optimizes embeddings to decrease sparsity and increase model resilience.

**Fig 2:**
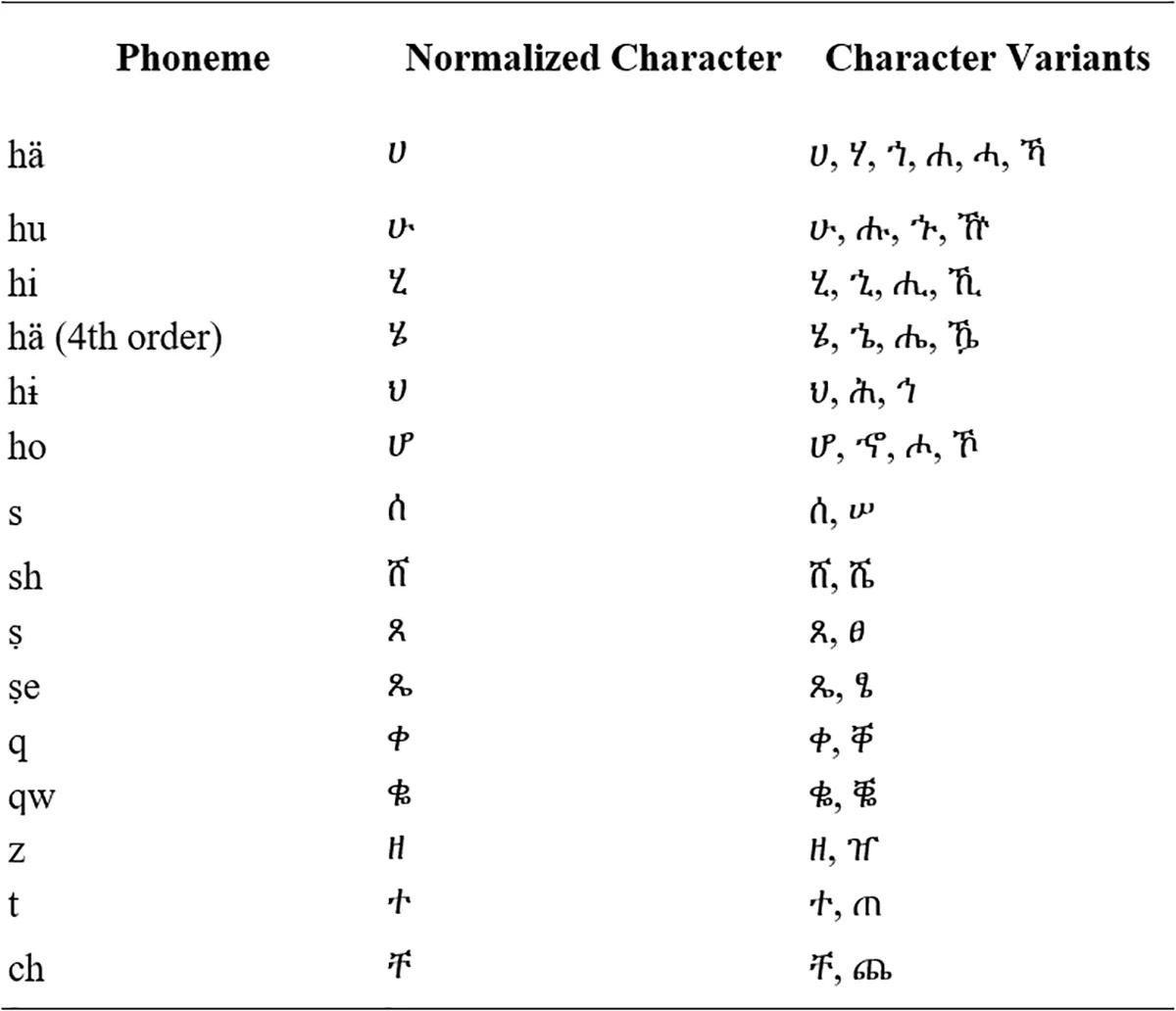
Text Normalization.

There was a class imbalance issue in the dataset, as suicidal posts are much less common than non-suicidal posts (Fig 3). To address this issue, weight-balancing procedures were used during model training to give the minority class higher weights [43]. The model is therefore encouraged to concentrate more on the underrepresented class, which improves its ability to recognize suicidal posts.

**Fig 3:**
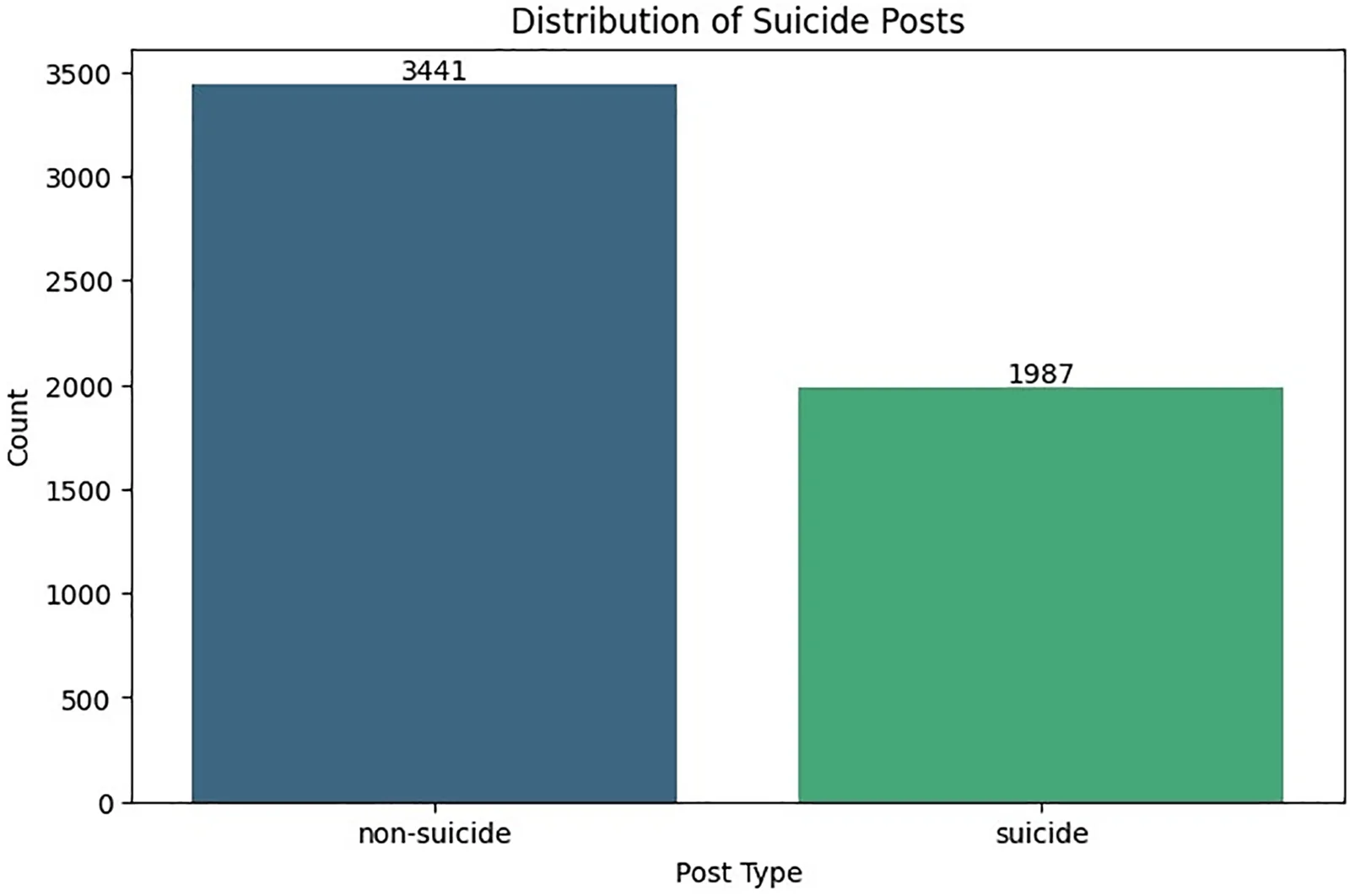
Distribution of the dataset.

### 3.2 Data annotation

Three domain experts used a wide, comprehensive keyword-guided annotation standard to annotate the dataset. The sample suicidal keywords were shown in Fig 4, and annotators considered semantic meaning, surrounding context, and the message’s intent rather than relying on individual keywords; each sentence was evaluated separately and classified as suicidal or non-suicidal. For example, expressions indicating a direct intention to harm oneself were classified as suicidal, whereas general expressions of sadness, frustration, or criticism without self-harm intention were classified as non-suicidal.

**Fig 4:**
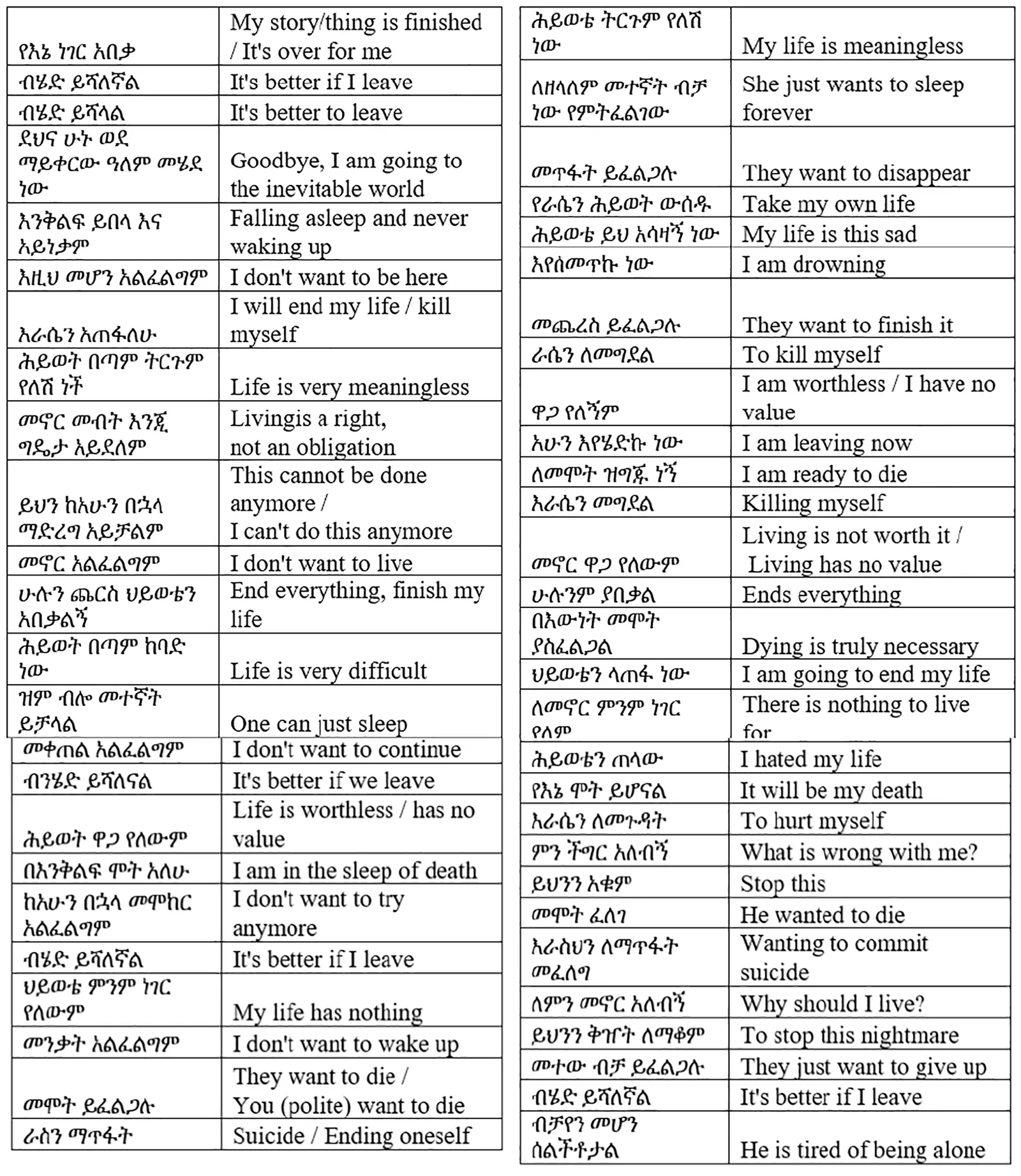
Sample suicidal keywords (Amharic-English).

The class-wise annotation consistency was assessed using observed agreement, as shown in Table 1, which produced 96.28 agreement for suicidal ideation and 96.89 agreement for non-suicidal cases. Cohen's Kappa was used to assess inter-annotator agreement [44], and the result was a perfect agreement of 0.93. Majority voting was used to determine the final label, and expert discussions were used to settle any outstanding disputes. The number of suicidal posts that did not contain any of the predefined keyword indicators was not recorded separately during annotation and therefore was not quantified in this study.

### 3.3 Tuning parameters

The hyperparameters of the suggested models are shown in Table 2. These were established by a thorough literature review and methodical empirical adjustment [45].

**Table 2:** Hyper-parameter selection of the proposed model.

Parameter	Search Space	Selected Value
Embedding Dimension	128, 256, 512	256
Activation function	ReLU, Sigmoid	Sigmoid
Loss function	Binary cross-entropy	Binary cross-entropy
Dropout Rate	0.1, 0.2, 0.3	0.3
Learning Rate	1e-3, 5e-4, 1e-4	1e-3 (0.001)
Epochs	5, 20, 25, 30	25
Optimizer	Adam, AdamW, SGD	Adam
Batch Size	16, 32, 64	32

## 4 Results

The identification of suicidal ideation in Amharic texts is thoroughly examined in this section. It uses common performance evaluation measures to assess the outcomes of machine learning, deep learning, and ensemble learning models. It also discusses the current research problems in suicidal ideation detection and evaluates pertinent literature.

As shown in Fig 5, baseline learning models including Logistic Regression (LR), Random Forest (RF), Support Vector Machine (SVM), and Naïve Bayes were first evaluated. The experimental results for the proposed models are summarized in Table 3 and Fig 6. Based on the experimental results, ensemble learning models achieved slightly higher observed performance than the individual deep learning models in suicidal ideation detection. Specifically, hard voting ensemble learning models achieved the highest accuracy (83.3%) and precision (86.55%). Among individual deep learning models, CNN-BiLSTM achieved the highest accuracy (81.45%), while the BiLSTM model achieved an accuracy of 81.4% and a nearly identical F1-score (80.8%), recall (77.6%), and precision (84.5%), indicating that bidirectional sequence learning effectively captures contextual information in Amharic texts.

**Table 3:** Model evaluation results.

Applied Models	Accuracy	Precision	Recall	F1-Score
Ensemble (hard voting)	0.832300	**0.865500**	0.784100	0.822500
Ensemble (soft voting)	0.828600	**0.852100**	0.804900	0.827600
Ensemble (Weighted voting)	0.828600	**0.852000**	0.806000	0.828100
Bi-LSTM	0.814091	**0.845508**	0.776094	0.808258
CNN-Bi-LSTM	0.814453	**0.847462**	0.774612	0.808263
CNN-LSTM	0.809290	**0.847620**	0.760741	0.800804
LSTM	0.807448	**0.850191**	0.753804	0.797651
CNN	0.806893	**0.844260**	0.760726	0.798892

**Fig 5:**
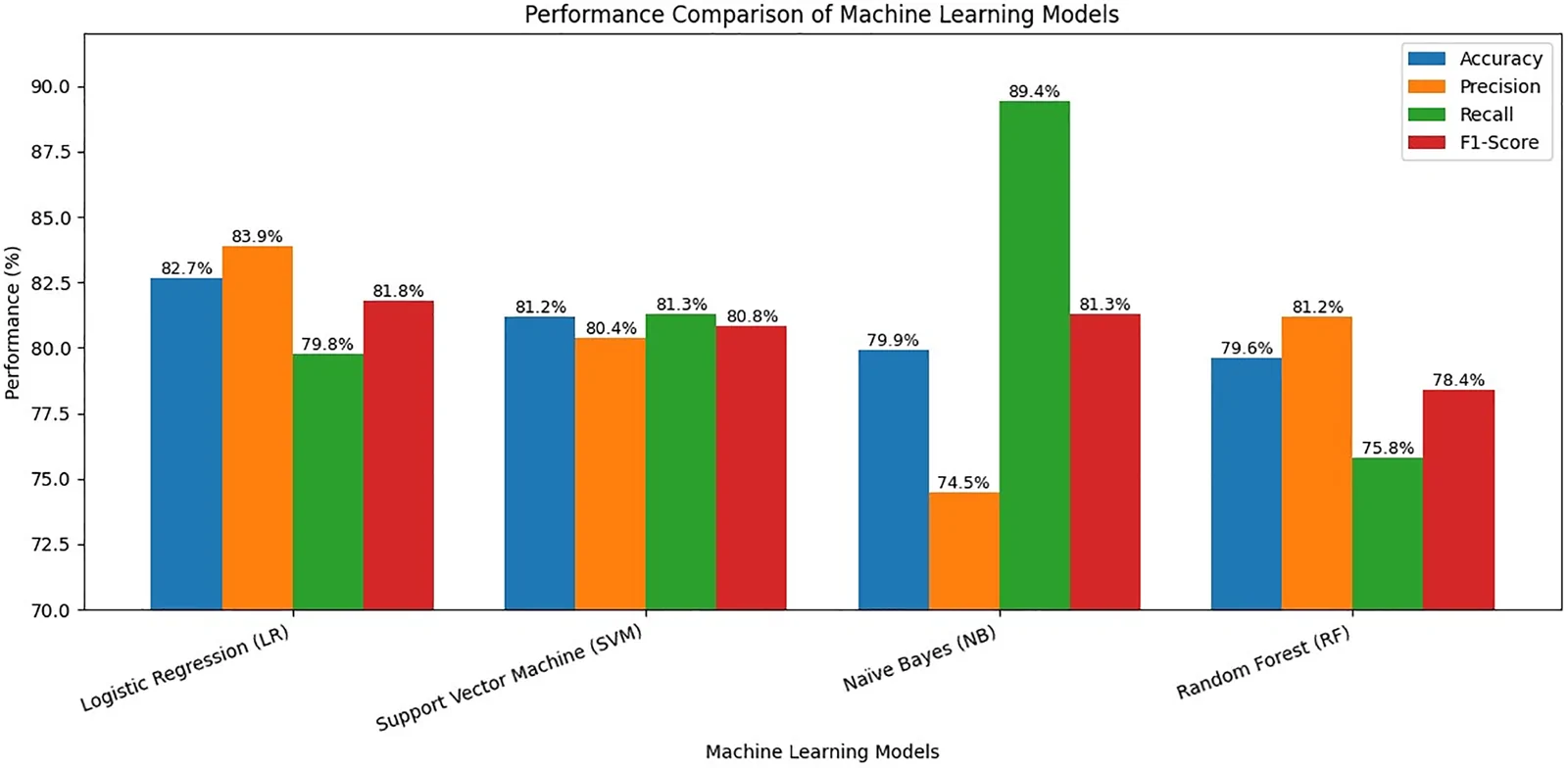
Experimental results of baseline machine learning models.

**Fig 6:**
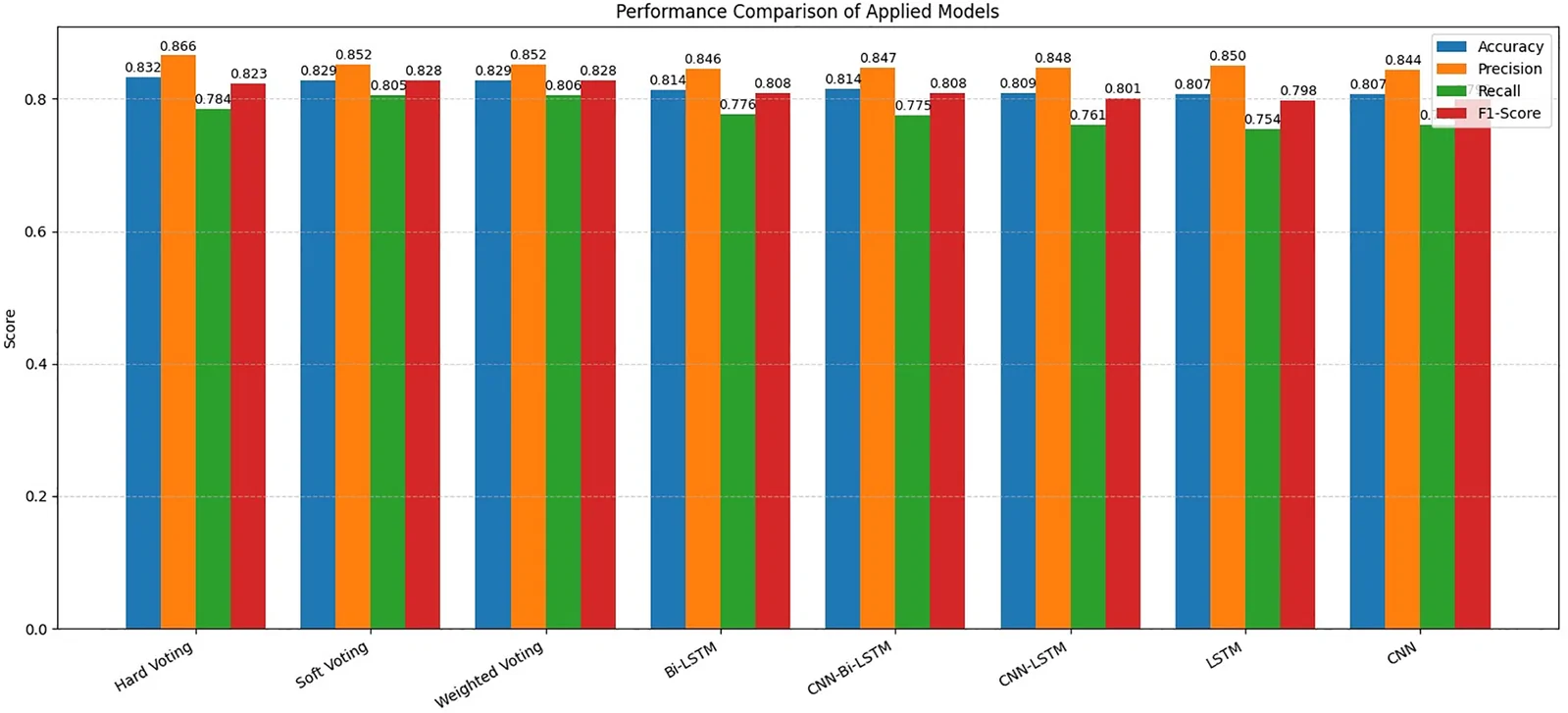
Experimental results of deep learning and ensemble models.

The CNN-LSTM, LSTM, and CNN models showed slightly lower performance, with accuracies ranging from 80.69% to 80.93%. Overall, the findings indicate that ensemble learning provides more robust and reliable predictions than standalone deep learning models by leveraging the complementary strengths of multiple classifiers.

As depicted in Figs 7 and 8, the confusion matrix obtained from 10-Fold cross-validation for the CNN and LSTM models shows that both models effectively distinguish between suicidal (class 1) and non-suicidal (class 0) content. The CNN model demonstrates a slight advantage in identifying suicidal instances, as indicated by fewer false negatives, whereas the LSTM model exhibits marginally better performance in correctly classifying non-suicidal content, reflected by a lower false-positive rate.

**Fig 7:**
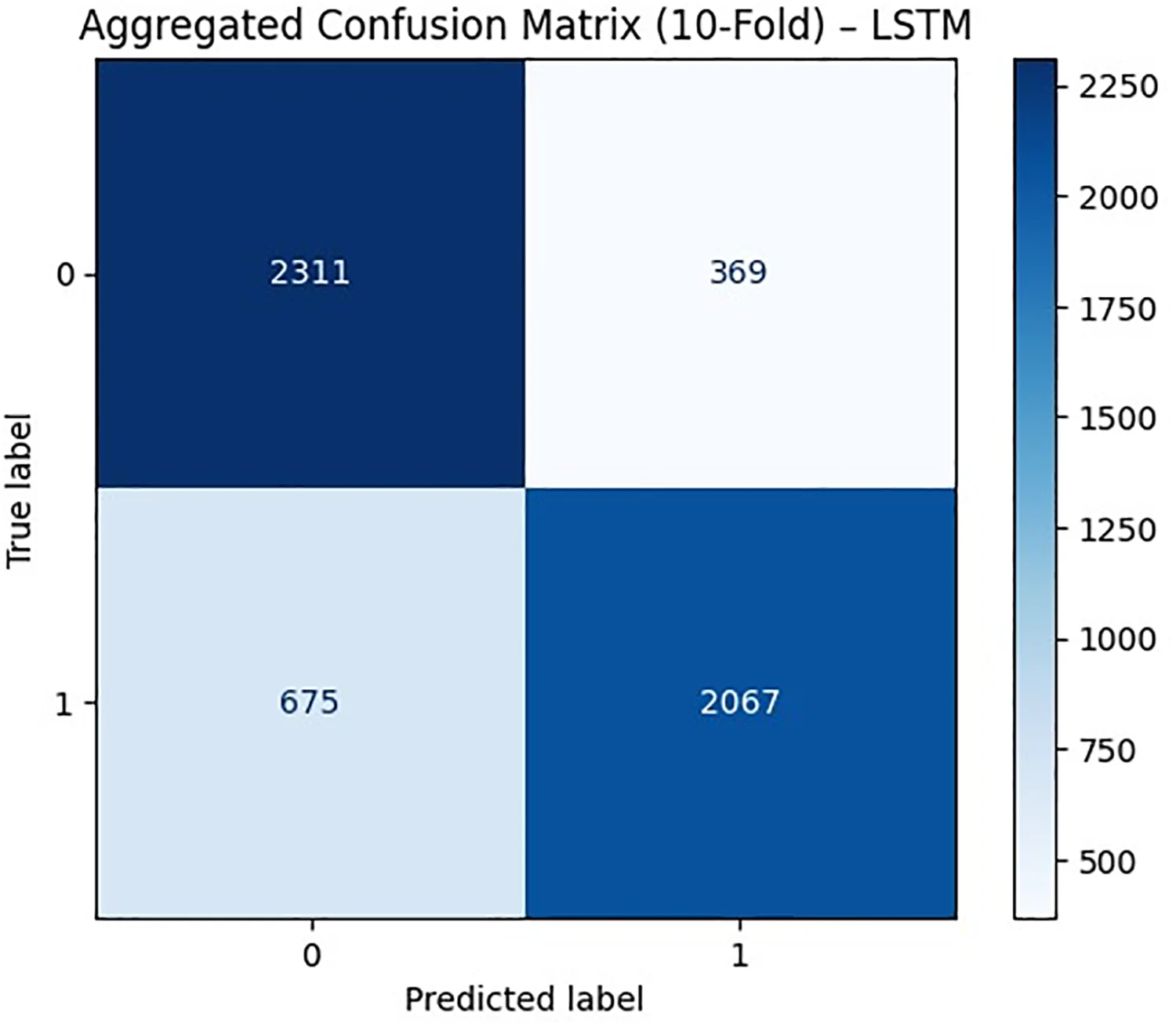
Confusion matrix of (10-Fold)-CNN model.

**Fig 8:**
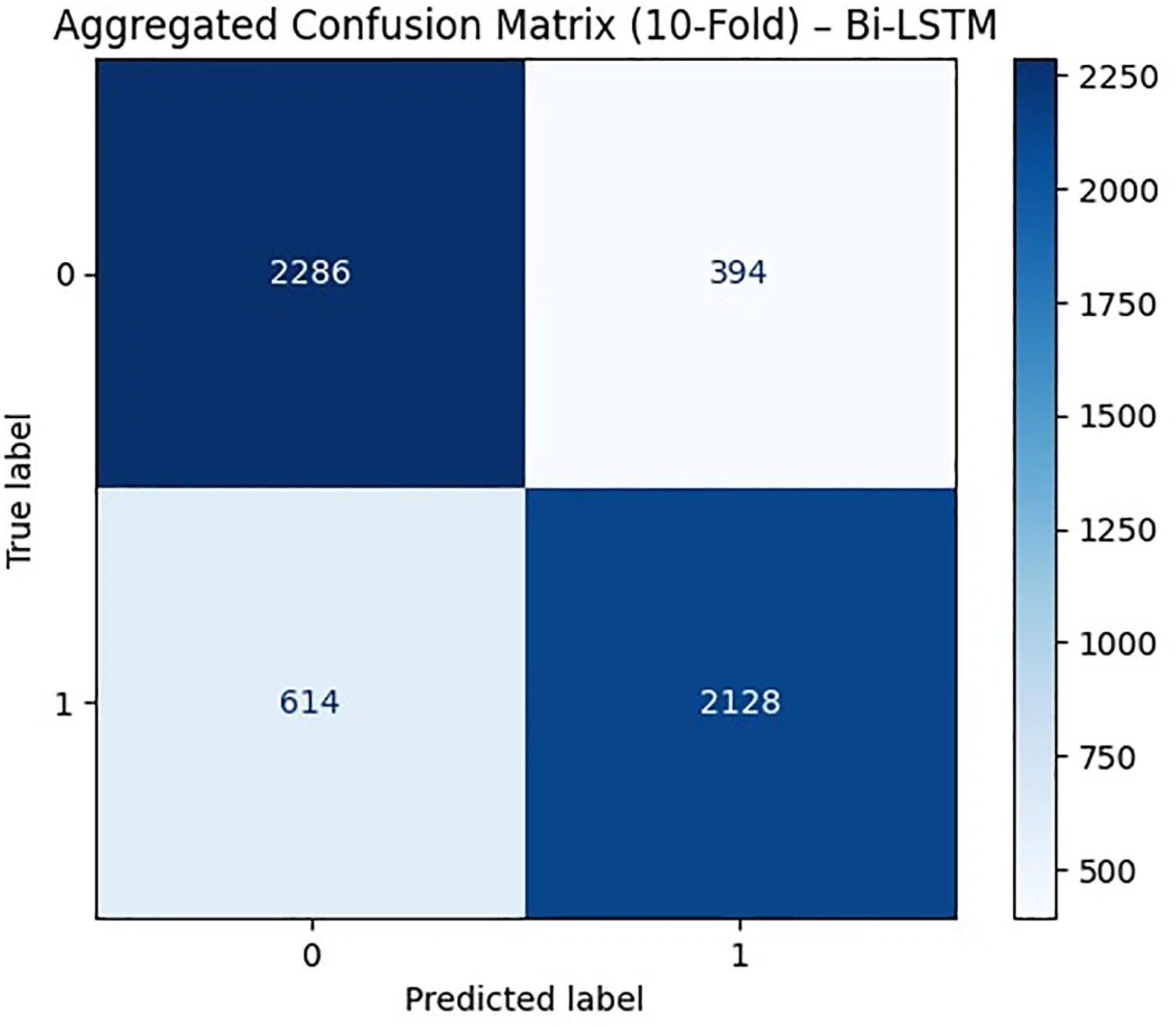
Confusion matrix of (10-Fold)-LSTM model.

As shown in Figs 9 and 10, the aggregated confusion matrix indicates that the Bi-LSTM model demonstrates superior sensitivity to suicidal content by correctly identifying a higher number of suicidal instances and producing fewer false negatives compared to the CNN-LSTM model. Similarly, Bi-LSTM lowers the critical risk of incorrectly identifying suicidal text as non-suicidal, but it produces more false positives. The CNN-LSTM model, on the other hand, did better at identifying non-suicidal content and generated fewer false positives, but it misses more suicidal content, which increases the risk in practical mental health applications. When it comes to identifying suicidal content, the Bi-LSTM model performs more consistently and clearly than the other models. The CNN-BiLSTM model's confusion matrix, which is shown in Fig 11, accurately detects the majority of suicidal cases while overlooking a moderate number of high-risk ones. Additionally, it correctly categorizes most texts that are not suicidal, with comparatively few false positives.

**Fig 9:**
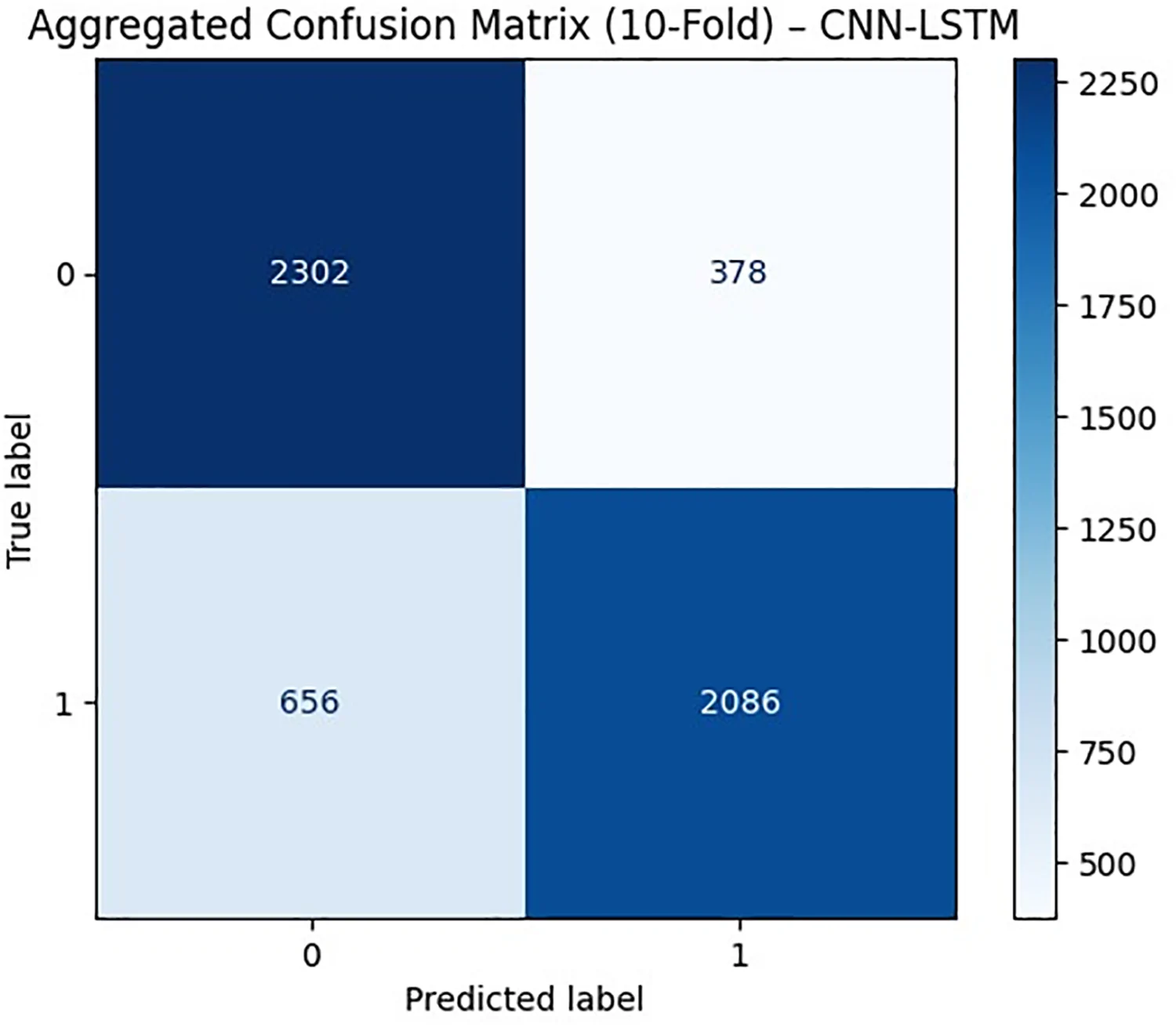
Confusion matrix of (10-Fold)-Bi-LSTM model.

**Fig 10:**
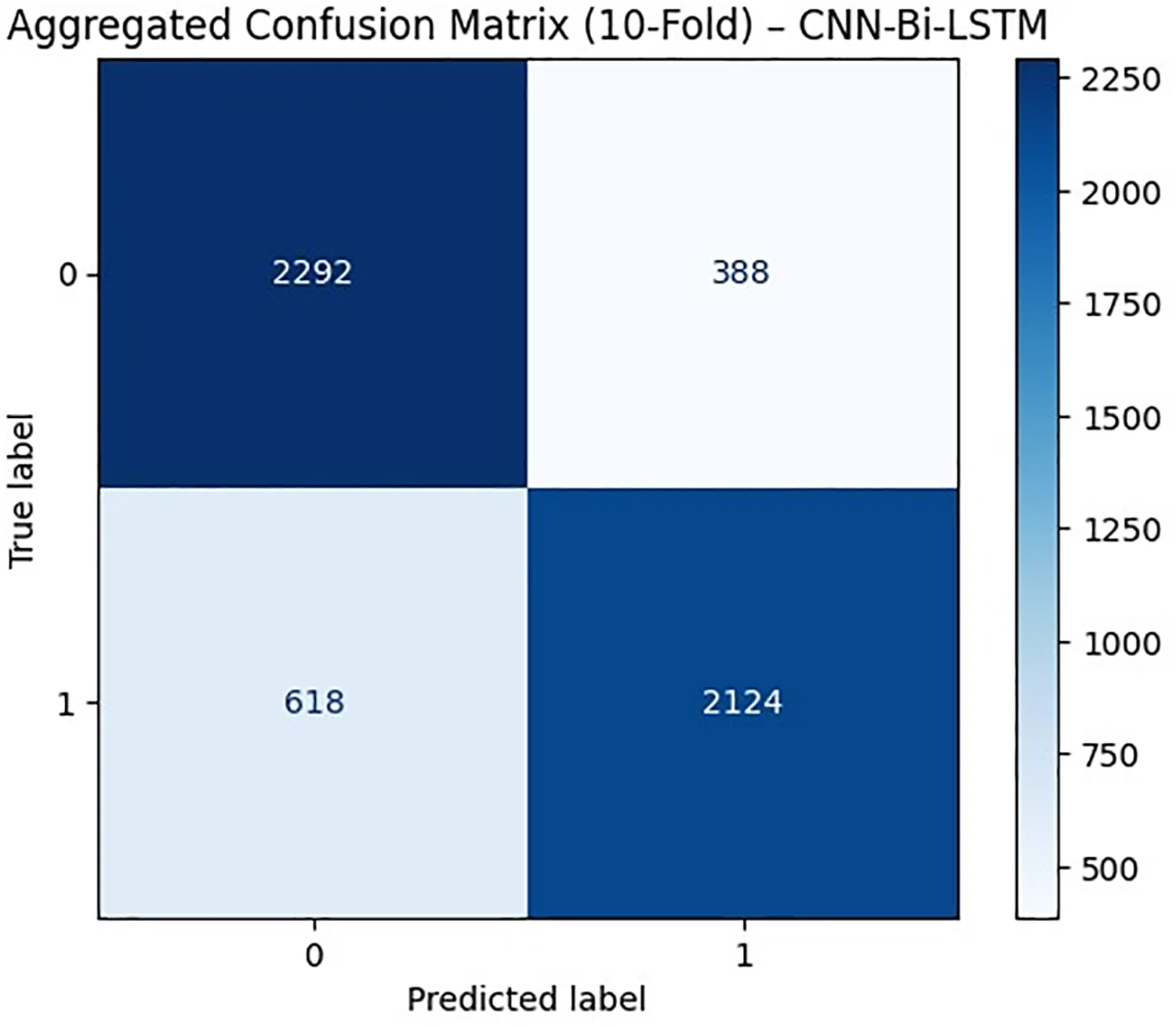
Confusion matrix of (10-Fold)-CNN-LSTM model.

**Fig 11:**
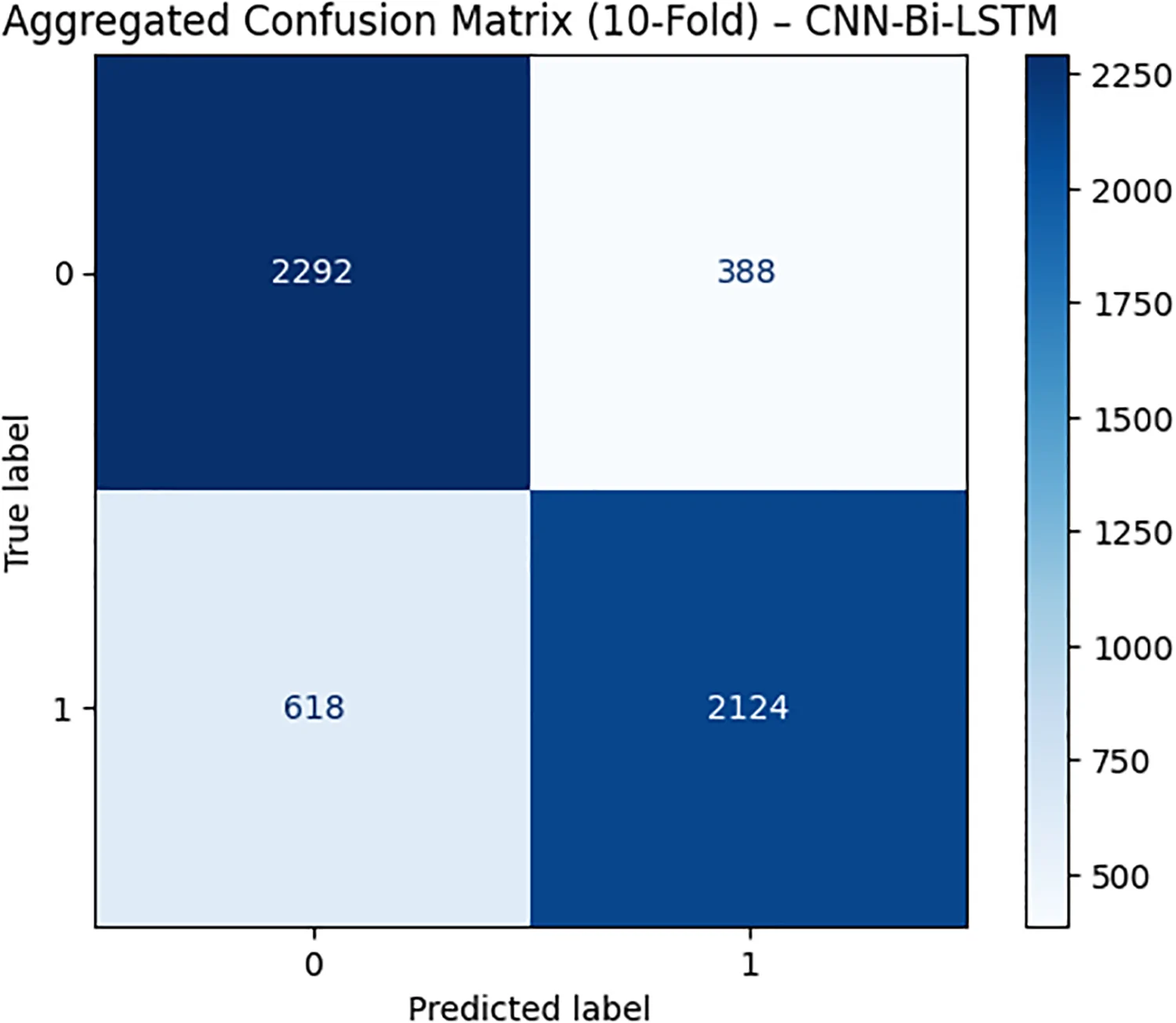
Confusion matrix of (10-Fold)-CNN-Bi-LSTM model.

As depicted in Fig 12, the detection performance of the ensemble learning models reveals critical insights. Hard voting demonstrates solid results but tends to overlook more instances of suicidal content. In contrast, soft voting improves sensitivity. However, the weighted voting model outperforms the others by accurately identifying the most critical suicidal instances while minimizing missed content, making more reliable and safety-focused approach.

**Fig 12:**
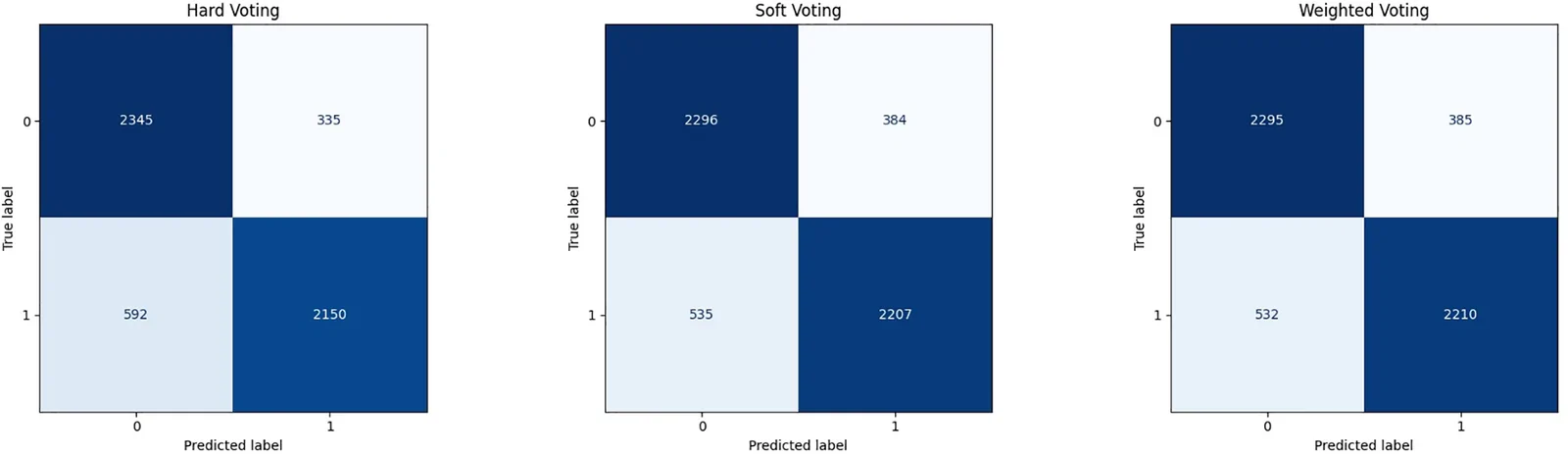
Confusion matrix of (10-Fold)-Hard, Soft, and Weighted Voting models.

As shown in Table 4 and Fig 13, all models achieved relatively high accuracy on both training and validation data, indicating a successful learning process. The ensemble learning models demonstrated a competitive generalization performance with relatively small differences between training and validation accuracies. Among evaluated models, the CNN model exhibited the largest training-validation accuracy gap, indicating comparatively greater susceptibility to overfitting, while ensemble learning models maintained stable performance on unseen data. The LSTM model achieved a balanced performance with a training accuracy of 84.7% and the highest validation accuracy (82.9%), indicating better generalization to unseen data. Bi-LSTM and CNN-BiLSTM models also demonstrated good generalization, with validation accuracies of 82.7% and 82.6%, respectively. The CNN-LSTM model had a high training accuracy (84.9%) but a lower validation accuracy (81.7%), indicating possible overfitting due to increased complexity. Ensemble models (hard voting, soft voting, and weighted voting) also performed well, with hard voting achieving a training accuracy of 85% and a validation accuracy of 82%, though their validation performance was generally lower than that of the best individual models. Overall, the LSTM model provided the most reliable performance due to its superior validation accuracy and balanced training behavior, while the CNN model showed strong learning but less generalization. Early stopping with a patience value of 2 was used to prevent overfitting, stopping training if validation performance did not improve for two consecutive epochs.

**Table 4:** Training and validation accuracy.

Learning Models	Training Accuracy	Validation Accuracy
Ensemble (hard voting)	0.851400	0.819900
Ensemble (soft voting)	0.828600	0.802100
Ensemble (Weighted voting)	0.848600	0.812000
Bi-LSTM	0.848147	0.827237
CNN-Bi-LSTM	0.845603	0.826059
CNN-LSTM	0.849106	0.817077
LSTM	0.846671	0.828586
CNN	0.857162	0.819570

**Fig 13:**
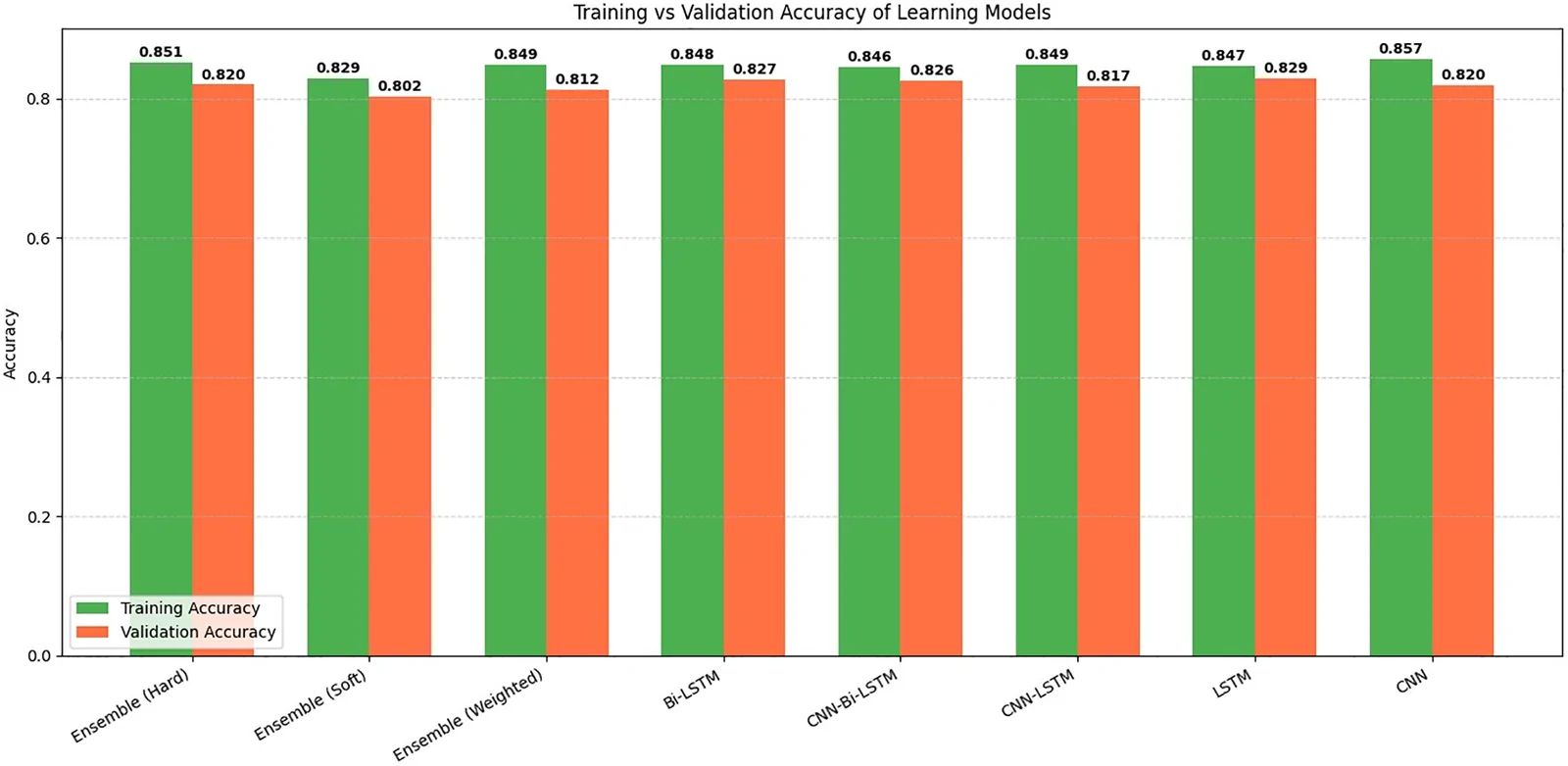
Training and validation accuracy of learning models.

As shown in Tables 5 and 6, most recent studies on suicidal ideation detection have largely concentrated on English-language social media data, especially from platforms like Reddit and Twitter, relying mainly on text-based inputs. In addition, advanced deep learning models like Convolutional Neural Networks (CNN), Long Short-Term Memory networks (LSTM), Bidirectional LSTM (BiLSTM), hybrid CNN-LSTM models, and transformer-based frameworks like BERT and GPT are gradually replacing conventional machine learning models like XGBoost and SVM. With accuracy and F1-scores ranging from 80% to 99.99%, these deep learning models show optimum performance.

**Table 5:** Summary table of related works, dataset, data type, techniques, and evaluation metrics used.

Ref.	Year	Language Used	Dataset Used	Data Types	Techniques Used	Evaluation Metrics in%	Key Contribution	Research Gaps
[13]	2022	English	Reddit (232,000 posts)	Text	TF-IDF, Word2Vec, XGBoost, CNN-BiLSTM	ACC = 95	CNN-BiLSTM achieved 95% accuracy, outperforming XGBoost, and shows DL effectiveness for early suicide detection	Limited to unimodal text data; lacks multimodal or multilingual analysis
[21]	2020	English	Reddit	Text	RF, SVM, NB, XGBoost, LSTM, CNN, CNN-LSTM	ACC = 93.8 Recall = 94.1 F1-score = 93.4 Precision = 93.2	CNN-LSTM achieved 93.8% accuracy and 93.4% F1-score, outperforming baseline models	Only textual data; lacks manual annotation and external dataset validation
[12]	2025	English	Suicide Detection, CEASEv2.0, SWMH	Text	Word Embedding, BERT, Transformer-based models	ACC = 99.6/ 70.0 F1-score = 97/ 75 / 68	Achieved state-of-the-art results (F1 = 0.97, Acc = 0.996 on (Suicide Detection datasets)	Lower performance on SWMH; data imbalance and noise issues; privacy and bias concerns
[22]	2025	English	Reddit, CEASE-v2.0	Text, Emotion	CoDyn-BMHSA-CNN	ACC = 98.2/ 97.4 Rec = 95.2/ 92.7 Precision = 96.8 / 94.1	Achieved 98.2% and 97.4% accuracy on two datasets; emphasized emotional analysis for early detection	Limited to emotion-based textual features; lacks multimodal or contextual analysis
[5]	2024	English	Reddit	Text	CNN-BiLSTM, GPT, BERT, RoBERTa, GPT-2	ACC = 94.5 (C-BiLSTM), 97.69 (GPT)	GPT achieved 97.69%, outperforming classical ML and DL models	Focused on textual Reddit data only; lacks generalization to other platforms
[19]	2025	English	Reddit	Text + Emojis (2D images)	VGG-16, ResNet-50, AlexNet	ACC = 99.99	Converted text and emoji into 2D images, achieving 99.99% accuracy	Model not validated across datasets; interpretability and generalization not tested
[24]	2021	English	Reddit	Text	LSTM-Attention-CNN (Ensemble)	ACC = 90.3 F1-score = 92.6	Classified suicide risk levels; achieved 90.3% accuracy and 92.6% F1-score	Lacks cross-platform validation and multimodal integration
[25]	2025	English	Kaggle (Depression dataset)	Text	BERT-BiLSTM + Knowledge Graph	ACC = 88.95 Recall = 93.81 F1-score = 89.55 Precision = 85.6	Achieved 88.95% accuracy, enhanced contextual understanding using knowledge graphs	Limited to depression (not ideation); single-source data
[26]	2025	English	Reddit, Twitter	Text	GWO+ Recursive Feature Elimination + SFS	ACC = 98 (Reddit), 97 (Twitter)	Achieved 98% (Reddit) and 97% (Twitter); improved dimensionality reduction	Focused on feature selection; lacks deep contextual or semantic modeling
[46]	2024	English	Reddit	Text + Metadata	Transformer + Metadata + Linguistic Features	Recall = 84.18 F1-score = 84.15 Precision = 84.26	Improved F1 from 72.59% to 84.15% using data augmentation (EDA)	Requires multimodal expansion and real-time adaptability
[41]	2024	English	Twitter	Text	GANs, CNN + FastText, LSTM + Fusion, Genetic Algorithms	ACC = 89.73 F1-score = 99 (Low), 98 (High Risk)	F1 = 0.99 (Low Risk), 0.98 (High Risk); showed the strength of hybrid models	Complex architecture; limited interpretability; lacks cross-dataset validation
[28]	2023	English	Twitter, Reddit	Text	XGBoost + EFASRI (TF-IDF, LSI, ARS, Topic Modeling)	ACC = 96.33	Achieved 96.33% accuracy; effective for multi-risk level classification	Relies heavily on feature engineering; limited adaptability to unseen data
[30]	2025	English	Twitter	Text	NLP + ML	ACC = 85 Recall = 83 Precision = 88	Achieved 85% accuracy and PR-AUC 93%; strong in high-risk detection	Limited dataset; no deep contextual or multimodal analysis

**Table 6:** Comparative analysis of the proposed model with the state-of-the-art used dataset, techniques, and evaluation metrics.

Ref.	Dataset Used	Techniques Used	Accuracy	Precision	Recall	F1-score
[19]	Reddit	VGG-16, ResNet-50, AlexNet	99.99	–	–	–
[12]	Suicide Detection, CEASEv2.0, SWMH	Word Embedding, Transformer (BERT)	99.6 / 70.0	–	–	97 / 75 / 68
[22]	Reddit, CEASE-v2.0	CoDyn-BMHSA-CNN	98.2 / 97.4	96.8 / 94.1	95.2 / 92.7	–
[26]	Reddit	Grey Wolf Optimization + RFE + Stepwise FS	98	97	–	–
[28]	Twitter, Reddit	XGBoost + EFASRI (TF-IDF, LSI, ARS, Topic Modeling)	96.33	–	–	–
[5]	Reddit	CNN-BiLSTM, GPT, BERT, RoBERTa, GPT-2	97.69 (GPT), 94.5 (C-BiLSTM)	–	–	–
[21]	Reddit	RF, SVM, NB, XGBoost, LSTM, CNN, CNN-LSTM	93.8	93.2	94.1	93.4
[46]	Reddit, Twitter	Transformer + Metadata + Linguistic Features	–	84.26	84.18	84.15
[24]	Reddit	LSTM-Attention-CNN (Ensemble)	90.3	–	92.6	–
[25]	Kaggle (Depression dataset)	BERT-BiLSTM + Knowledge Graph	88.95	85.66	93.81	89.55
[41]	Twitter	GANs, CNN + FastText, LSTM + Fusion, Genetic Algorithms	89.73	99 (Low), 98 (High Risk)	–	–
[30]	Twitter	NLP + ML	85	88	83	–

The proposed model integrates and evaluates five deep learning architectures: CNN, LSTM, Bi-LSTM, CNN-LSTM, and CNN-BiLSTM, along with ensemble learning approaches. It achieved over 81% across all evaluation metrics, including Accuracy, Precision, Recall, and F1-score, outperforming existing methods in low-resource language settings. This improvement results from combining convolutional and recurrent layers to learn both phrasal and sequential text features, supported by effective preprocessing and optimized hyperparameters.

### 4.1 Summary of related works

As presented in Table 5, and Fig 14, the summary of the existing state-of-the-art and literature review with quantitative comparative analysis results reveals that there were comparably similar accuracy results in both deep learning models and transformer-based learning models on textual and multimodal data types. Further, it was observed that there was limited research effort on multimodal data content, and even limited research effort on low-resource languages on both textual and multimodal content, and no standard dataset.

**Fig 14:**
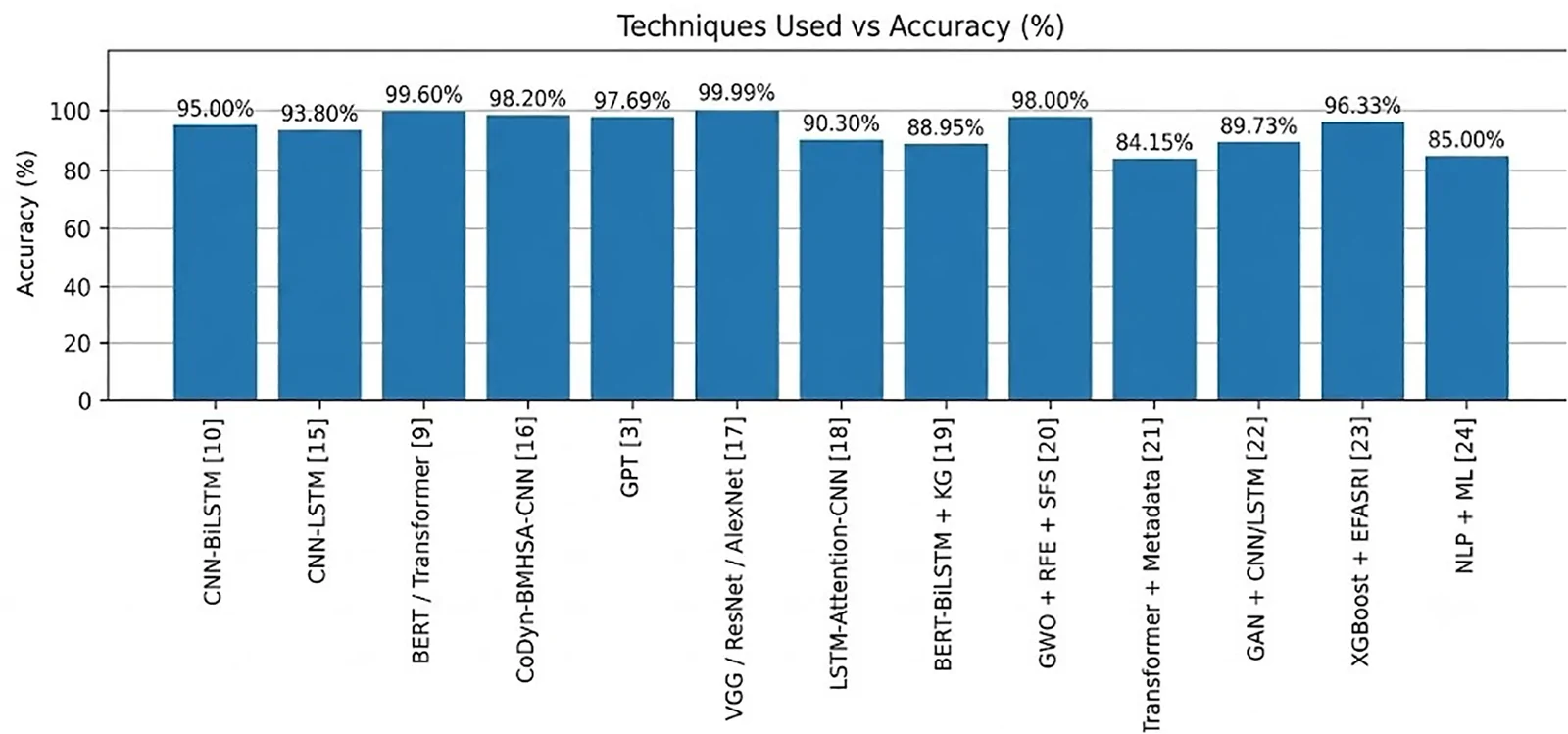
Summary of the existing state-of-the-art related works (accuracy and techniques used).

As illustrated in Table 6, the proposed model demonstrated solid and consistent performance, showing lower accuracy scores ranging from approximately 80% to 81%, compared to state-of-the-art models’ accuracy on benchmark datasets such as Reddit, Twitter, or CEASE-v2.0. The model used a unique custom dataset, which was collected from social media platforms and was likely more challenging, messier, and contained more informal language and mixed dialects. In general, the results were developed on five different deep learning models (CNN, LSTM, Bi-LSTM, CNN-BiLSTM, CNN-LSTM), which show that the performance is very stable, ranging from 80% to 81% accuracy and F1-score from 79% to 80%. This suggests that the feature engineering (FastText, GloVe, Word2Vec) was effective, and that the models are not overfitting on the small dataset. Furthermore, the model exhibits a respectable balance between recall ranging from 75% to 78%), precision (ranging from 84% to 85%), and F1-score from 79% to 80%). For linguistic localization in the real world for suicide risk detection, this performance shows that the model was not skewed toward predicting one particular class, which is significant and practical.

## 5 Discussion

The findings of this study demonstrate that deep learning and ensemble learning approaches are highly effective in identifying suicidal thoughts in Amharic social media texts, highlighting their potential for application in low-resource language environments. The LSTM model achieved the most balanced performance among all models, with an accuracy of 80.7% and an F1-score of 79.7%. The proposed ensemble learning model, which integrates CNN, CNN-LSTM, CNN-Bi-LSTM, LSTM, and Bi-LSTM models, achieved a marginal improvement in prediction performance. This underscores the methodological advantage of combining convolutional and recurrent networks, as hybrid models can capture both long-term sequential dependencies and localized textual features. Notably, the proposed model performs comparably to models developed for English-language datasets, despite being applied to a low-resource language.

This accomplishment is attributed to comprehensive text preprocessing, the use of semantically rich embedding-based feature representations such as Word2Vec, GloVe, and FastText, and systematic hyperparameter optimization. By developing and publicly releasing the first Amharic-language dataset for suicidal ideation detection, this study addresses a significant resource gap in mental health research. The most frequently used Amharic phrases expressing suicide intent are illustrated in the word cloud visualizations for suicidal and non-suicidal ideation (Figs 15 and 16). Prominent words which reflect themes of isolation, pain, and hopelessness. The relative frequency of each term provides insights into distress-related linguistic patterns, offering valuable cues for identifying risk signals and informing targeted intervention strategies.

**Fig 15:**
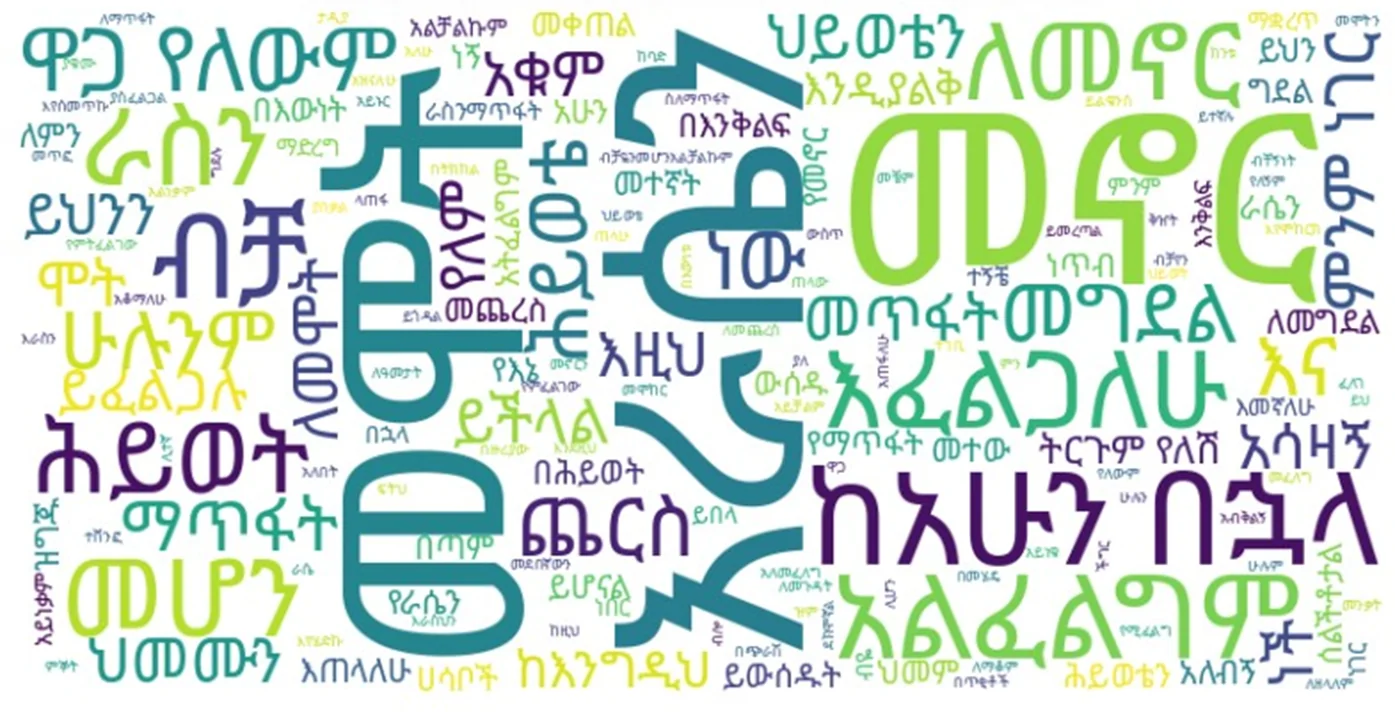
Word clouds on suicidal ideation.

**Fig 16:**
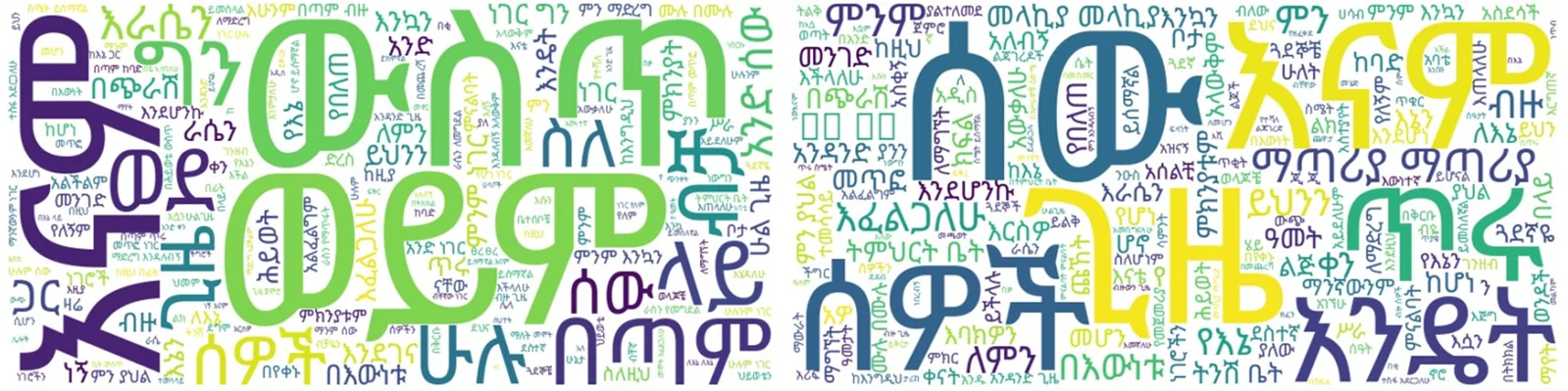
Word clouds on non-suicidal ideation.

In general, the findings suggest that hybrid and ensemble learning approaches are particularly effective at capturing complex verbal and emotional cues associated with suicidal thoughts. Beyond the comparative performance of evaluated models, the findings have practical significance for developing language-specific early identification of individuals at risk and effective social media monitoring. In addition, the model automatically flags Amharic posts containing linguistic indicators associated with suicidal ideation and prioritizes them for subsequent assessments. However, challenges remain, such as the limited amount of data and the need to incorporate more context-rich or multimodal elements (e.g., memes, images, audio, and video). Considering these shortcomings, the study's findings provide preliminary evidence for the potential of AI in underrepresented language mental health surveillance technologies.

Although the proposed study provides a comprehensive comparison of conventional machine learning, deep learning, and ensemble learning models, it does not include multilingual transformer-based architectures such as mBERT, XLM-RoBERTa, AfriBERTa, and AfroXLMR. These pre-trained models exhibit contextual embeddings from large multilingual corpora and have demonstrated superior performance on numerous low-resource NLP tasks through transformer learning and contextual understanding. The exclusion of these transformer models represents a limitation of this study. Embeddings were trained from scratch using collected Amharic data, which has a relatively limited corpus size compared with pre-trained larger multilingual corpora. In addition, vocabulary size and out-of-vocabulary statistics were not recorded during preprocessing.

Future work will therefore investigate transfer learning and cross-lingual fine-tuning using multilingual transformer models to determine whether contextual representations further improve suicidal ideation detection in Amharic social media. Furthermore, comprehensive analysis of vocabulary coverage and out-of-vocabulary (OOV) words will be conducted to quantify lexical representation quality and evaluate their impact on model robustness, generalization, and predictive performance. External validation on temporally and platform-independent datasets will also be undertaken to assess robustness and real-world applicability.

## 6 Conclusion and future work

Amharic social media posts with suicidal thoughts are difficult to identify because of their limited linguistic resources and processing models. By developing the first adaptable deep learning model for detecting suicidal ideation in Amharic social media content, this work addresses a significant research gap in low-resource languages. To support this, a dataset consisting of 5,428 samples was developed, including 1,987 Suicidal and 3,441 Non-Suicidal instances, and a robust unimodal classifier. The other key contribution was the creation of a specialized 111-word indicator, incorporating 985 commonly used stop words, which enhances the model's semantic understanding. Moreover, the study conducted a thorough investigation of multiple machine learning, deep learning, and ensemble learning model, systematically evaluating their performance in suicidal ideation detection for classification tasks. The maximum accuracy of ~83% was attained by ensemble learning, approximately 81.4% accuracy by BiLSTM, CNN-LSTM at approximately 80.9%, and CNN-Bi-LSTM at approximately 81.4% based on experimental data. The reported experimental results represent internal validation only and should not be interpreted as evidence of generalization to an independent dataset collected at a different time or from other social media platforms. This suggests that hybrid and ensemble models perform better for classification. However, for better semantic language understanding of long dependencies with respect to special word indicators, deep learning was better than ensemble learning. These contributions advance natural language processing for low-resource languages like Amharic and lay the groundwork for effective suicidal ideation detection on social media. In general, this study contributes significantly to the fields of computational linguistics, mental health informatics, and low-resource NLP by providing both a methodological framework and a new dataset for future research. It is recommended that future research adopt a fusion model, emojis, multimodal, and multilingual approaches, supported by scalable annotated datasets, to further improve suicidal ideation detection within Ethiopia’s digital landscape. Future studies should also benchmark multilingual pretrained transformer models, including mBERT, XLM-RoBERTa, AfriBERTa, and AfroXLMR, to evaluate the benefits of contextual multilingual representations and cross-lingual transfer learning for Amharic suicidal ideation detection.

Furthermore, generative AI and large language models (LLMS) have gained global attention across various domains; our review of available literature and resources suggests limited evidence of their application within the Ethiopian context, particularly due to the scarcity of publicly available contextual datasets. Hence, it is imperative that future studies address this gap.

The abbreviations and notations employed in this study are listed in Table 7

**Table 7:** Abbreviations and Notations.

The abbreviations and notations used in this study.	
AI	Artificial Intelligence
Bi-LSTM	Bidirectional LSTM
CNN	Convolutional Neural Network
DL	Deep learning
DRL	Deep Reinforcement Learning
DTL	Deep Transfer Learning
GAI	Generative Artificial Intelligence
GANs	Generative Adversarial Networks
GCNs	Graph Convolutional Networks
GRU	Gated Recurrent Unit
LSTM	Long Short-Term Memory
ML	Machine Learning
MLP	Multi-Layer Perceptions
NLP	Natural Language Processing
RNN	Recurrent Neural Networks
SFS	Step-wise feature selections
