# Supplementary material for: A comparative analysis of machine learning, deep learning, and ensemble learning models for suicidal ideation detection on social media
Source: PLOS Ment Health. 2026 Oct 22;3(10):e0000721. doi: 10.1371/journal.pmen.0000721 (PMC13690196; doi:10.1371/journal.pmen.0000721)
Supplement: Supplementary file 1 [file pmen.0000721.s003.pdf]

Dear Editor,

Thank you very much for your email and for the valuable feedback provided by the reviewers regarding our manuscript entitled "**A Comparative Analysis on Suicidal Ideation Detection Using Machine Learning, Deep Learning, and Ensemble Learning Models on Social Media,"** Manuscript Number**: PMEN-D-26-00351R2**

We sincerely appreciate the time and effort dedicated by the reviewers and the editorial team in evaluating our work. We are pleased that the manuscript was considered valuable, and we highly appreciate the insightful suggestions provided to further improve the quality of the paper.

We acknowledge that the manuscript requires Minor revisions, and we are committed to carefully addressing each of the editor's and reviewers' comments. By considering this, we have carefully revised each comment.

The manuscript has been thoroughly revised in accordance with the feedback provided. A point-by-point response to the comments has been prepared and included in the revised submission, clearly indicating the changes made using **RED FONT**.

Thank you once again for your consideration and guidance. We look forward to hearing from you.

Yours sincerely,

Andualem Woldegiorgis (Corresponding author)

(On behalf of all co-authors)

Editor comments:

1. To check the characters in Tables 1 and 2.

Response: Thank you very much. We sincerely appreciate the opportunity to revise the manuscript. In response to the comment regarding the characters in **Tables 1 and 2**, we carefully reviewed the character representation and formatting. To ensure that all Amharic characters and their corresponding content are displayed correctly and consistently, we have **converted Tables 1 and 2 into images** in the revised manuscript.

In addition, we carefully checked **all tables and figures/images throughout the manuscript**, including their **numbering, labels, captions, and in-text references**, and corrected any inconsistencies identified. Corrected on page (11)

2. Regarding ORCID:

We confirm that all coauthors have verified their ORCID iDs in the PLOS submission system.

If there is any error, discrepancy, or remaining issue with the ORCID information or its verification in the submission system, we would be grateful if you could kindly let us know so that we can address it promptly.

We carefully reviewed the reference list and confirm that **no retracted papers are included** in the revised manuscript. We also checked the references for completeness and accuracy and made the necessary corrections
