# Supplementary material for: A comparative analysis of machine learning, deep learning, and ensemble learning models for suicidal ideation detection on social media
Source: PLOS Ment Health. 2026 Oct 22;3(10):e0000721. doi: 10.1371/journal.pmen.0000721 (PMC13690196; doi:10.1371/journal.pmen.0000721)
Supplement: Supplementary file 2 [file pmen.0000721.s002.docx]

Dear Editor,

Thank you very much for your email and for the valuable feedback provided by the reviewers regarding our manuscript entitled “**A Comparative Analysis on Suicidal Ideation Detection Using Machine Learning, Deep Learning, and Ensemble Learning Models on Social Media,”** **Submission ID:** f48d4171-7b29-4b90-92e2-88b34cafdbb2

We sincerely appreciate the time and effort dedicated by the reviewers and the editorial team in evaluating our work. We are pleased that the manuscript was considered valuable, and we highly appreciate the insightful suggestions provided to further improve the quality of the paper.

We acknowledge that the manuscript requires Major revisions, and we are committed to carefully addressing each of the editor's and reviewers’ comments. By considering this, we have carefully revised each comment.

The manuscript is thoroughly revised in accordance with the feedback provided and ensures that all concerns are properly resolved. A detailed, point-by-point response to the reviewers’ comments has been prepared and included in the revised submission, clearly indicating the changes made using **RED FONT**.

Thank you once again for your consideration and guidance. We look forward to hearing from you.

Yours sincerely,

Andualem Woldegiorgis (Corresponding author)

(On behalf of all co-authors)

**Editor comments:**

1. In your Methods section, please include additional information about your dataset and ensure that you have included a statement specifying whether the collection and analysis method complied with the terms and conditions for the source of the data.

Response: Thank you for the valuable suggestion. We have added further details about the dataset, including its source and collection procedure, and clarified the data collection and analysis, which was highlighted in RED FONT (Pages 8,9,10 and 11)

2. Please note that PLOS Mental Health has specific guidelines on code sharing for submissions in which author-generated code underpins the findings in the manuscript. In these cases, we expect all author-generated code to be made available without restrictions upon publication of the work. Please review our guidelines at https://journals.plos.org/mentalhealth/s/materials-and-software-sharing#loc-sharing-code and ensure that your code is shared in a way that follows best practice and facilitates reproducibility and reuse.

Response: Thank you for the clarification. We have reviewed code-sharing requirements and made all author-generated code available without restriction, with the repository details provided in the revised manuscript

3. Please provide separate figure files in .tif or .eps format.

For more information about figure files please see our guidelines:  LINK

https://journals.plos.org/digitalhealth/s/figures

https://journals.plos.org/mentalhealth/s/figures 
https://journals.plos.org/mentalhealth/s/figures#loc-file-requirements

Response: Thank you for the guidance. We have provided all figures as separate files in the required .tif formats and ensured that they comply with the PLOS figure-file requirements.

4. Please provide a complete Data Availability Statement in the submission form, ensuring you include all necessary access information or a reason for why you are unable to make your data freely accessible. If your research concerns only data provided within your submission, please write "All data are in the manuscript and/or supporting information files" as your Data Availability Statement.

Response: Thank you for the guidance. We have provided a complete Data Availability Statement in the declaration section, highlighted in RED FONT (Page 27)

**Reviewer 1 comments:**

1. The manuscript would benefit from careful language editing to improve grammar, sentence structure, and readability. There are several instances where awkward phrasing affects clarity. Some examples are provided below.
   In the Abstract "From the experimental results, the LSTM model outperformed and optimally the other models" should be revised to: "Based on the experimental results, the LSTM model performed optimally, outperforming the other models." In the introduction, "According to the WHO, more than 800,000 persons die by suicide each year worldwide, which is equivalent to one death every 40 seconds."
   Consider revising to: "According to the World Health Organization, more than 800,000 people worldwide die by suicide each year, equivalent to one death every 40 seconds."

Response: Thank you very much for your careful evaluation and constructive comments. The abstract has been corrected, and the introduction has been revised and highlighted in RED FONT (Pages 1 and 2). The reported performance now matches Table 5:

2. The introduction provides useful background on suicide and social media; however, it does not adequately establish the specific research gap that motivates this study. The research questions appear rather abruptly at the end of the introduction before the reader has developed a clear understanding of the existing literature and its limitations. Although the manuscript states that research is limited in Ethiopia, this alone is not a sufficiently compelling justification. The introduction would be strengthened by explaining why existing models developed for high-resource languages are insufficient; the linguistic and cultural challenges associated with Amharic; and why these challenges justify the development of a language-specific model. I also suggest integrating the current "Related Works" section into the introduction to produce a single, coherent narrative that culminates naturally in the research questions.

Response: Thank you for this valuable suggestion. We have substantially revised the Introduction to provide a stronger motivation for the study, which was highlighted in RED FONT (Pages 2 and 3)

3. While the development of an Amharic-language suicidal ideation dataset appears to be one of the manuscript's primary contributions, this is not explicitly articulated. The manuscript would benefit from a concise statement explaining exactly how this work advances the existing literature beyond previous studies, rather than leaving the novelty for the reader to infer.

Response: Thank you for this valuable suggestion. We have revised the Introduction to explicitly state the novelty and primary contributions of this study, which was highlighted in RED FONT (Pages 2 and 3)

4. Please carefully review the reference list to remove duplicate entries and ensure that only the final published versions are cited. 12 and 13 appear to be the same paper, as does 7 and 22.

Response: Thank you for identifying these inconsistencies. We have carefully reviewed the entire reference list to remove duplicate entries and ensure consistency. (Pages 28- 32)

5. Throughout the Related Works section, studies are primarily introduced using phrases such as "A study in [citation]". It would improve readability to refer to studies using the first author's surname (e.g., "Basyouni et al."). More importantly, the section is largely descriptive rather than analytical. While previous studies are summarised, there is little critical evaluation of the literature or discussion of how the existing evidence informs and motivates the present study. The authors should synthesise the literature by identifying common trends, methodological limitations, and unresolved research gaps.

Response: Thank you for this constructive comment. We have thoroughly revised the Related Works section to improve both readability and critical analysis, which was highlighted in RED FORT in Section 2. Related Works.

6. The stated research questions are not fully aligned with the methodology employed. In particular, RQ1 and RQ3 are review-oriented questions that would be more appropriate for a systematic or narrative review. However, the manuscript presents an experimental machine learning study. The research questions should instead focus on evaluating the proposed models and assessing their performance on the Amharic dataset.

Response: Thank you for this valuable observation. We agree that the original research questions were more appropriate for a literature review than for an experimental machine learning study. Accordingly, we have revised the research question RQ1 to align with the study's objectives and methodology.

7. Please clarify what is meant by the phrase "methodical approach" in the opening sentence of Section 3 (Materials and Methods). The terminology is vague and should be explained or replaced with more precise language.

**Response:** Thank you for this very important and clarity-related comment, which is highly appreciated. In response to your comment, we have revised and replaced "methodical approach" with "data-driven approach" to more accurately describe the research methodology and highlighted it in RED FONT (Page 8).

8. The manuscript occasionally refers to itself as conducting a "meta-analysis", which appears inaccurate. For example: "After identifying the problem, a review of the literature was conducted..." While the literature search appears systematic, the manuscript does not present the defining characteristics of a systematic review or meta-analysis (e.g., predefined eligibility criteria, PRISMA methodology, quality assessment, or quantitative synthesis). Instead, this section appears to present a narrative synthesis of previous work. Please clarify the purpose of the literature review and avoid referring to it as a meta-analysis unless a formal meta-analytic methodology has been undertaken.

Response: Thank you for this important clarity-related comment. We agree that the term **"meta-analysis"** was inaccurate for the scope of this study. Corrected and revised to: “To establish the research context and identify existing knowledge gaps, a review of the literature was conducted.

9. The manuscript provides a detailed description of data cleaning and annotation but gives relatively little information regarding data collection. Please clarify or explain why the following cannot be specified: the period during which Facebook and Telegram posts were collected; the specific groups or pages used (where appropriate); the scraping procedure; the inclusion and exclusion criteria used during data collection.

**Response:** Thank you for your comment. In response to your comment, it was appended and highlighted in RED FONT (Page 9).

10. Please clarify how suicidal and non-suicidal posts were operationally defined during annotation. Although inter-rater agreement is reported, readers would benefit from additional information regarding: annotation guidelines; decision criteria; examples of annotated posts; and how semantic context was interpreted during the annotation process.

Response: Thank you for the valuable suggestion. We have expanded the annotation description by adding the operational definitions of suicidal and non-suicidal classes, annotation guidelines, decision criteria, and an explanation of semantic context interpretation. It was appended and highlighted in RED FONT (Pages 8,9,11).

11. Was there a reason why more recent transformer-based multilingual baselines (e.g., multilingual BERT or XLM-RoBERTa) were not included? Given their widespread use in multilingual NLP, it would be useful to justify their omission or discuss this as a limitation.

Response: Thank you very much for your valuable and insightful comments, which have significantly helped us improve and strengthen the manuscript. We agree that multilingual transformer models represent the current state-of-the-art for many text classification tasks. In the revised manuscript, we have expanded the discussion of transformer-based approaches in the Related Works section and acknowledged their importance for suicidal ideation detection. Since the primary objective of this study was to conduct a comparative analysis of conventional machine learning, deep learning, and ensemble learning methods using the first publicly available Amharic suicidal ideation dataset, transformer-based models were considered beyond the scope of the current study.

The newly added content has been appended and highlighted in Section 2, Related Works, Section 4, and Section 5, Conclusion and Future Works RED FONT (Pages 6, 23, and 25)

12. Parts of the Results section contain material that would be more appropriate within the Introduction or Discussion. For example, the discussion comparing recent literature on English-language datasets and the evolution from conventional machine learning models to transformer-based models represents background literature rather than experimental findings. Consider relocating this material to improve the overall structure of the manuscript.

Response: Thank you very much for your valuable and insightful comments. The results section now focuses specifically on the **findings of the reviewed studies**, improving the manuscript’s clarity and structure, as appended and highlighted in RED FONT (Pages 12 and 23)

13. The Discussion currently reads largely as a repetition of the Results section. The authors could expand the discussion by: discussing the practical significance of the findings and explaining how the proposed framework advances current knowledge. In particular, the intended application of the model should be clarified. Is it intended for early identification of individuals at risk; population-level surveillance; clinical decision support; or social media monitoring? Discussing the practical applications, alongside the ethical implications of deploying such models, would considerably strengthen the manuscript.

**Response**: Thank you for this valuable comment. We agree and have revised it to clarify the practical significance and intended application of the proposed model, which was highlighted in RED FONT (Page 24).

14. Given that many of the reported performance differences are relatively small, these claims of one model "outperformed", "performed optimally", or was "superior" to the others should either be supported with appropriate statistical analyses or expressed more cautiously.

Response: Thank you for this valuable comment. We agree that some performance differences are relatively small. We have revised the manuscript to use more cautious language and highlighted it in RED FONT throughout the manuscript

15. Although the manuscript states that publicly available social media data were used, the ethical considerations could be strengthened. As a reader, I was curious about whether additional safeguards were implemented before publicly releasing the dataset. Given the sensitive nature of suicide-related data, further discussion is warranted.

**Response**: Thank you very much for this important suggestion. We have added a separate “Declarations” section in the revised manuscript, and we have added links to the publicly available datasets in both the Data Availability Statement (DAS), Ethical Approval, and the relevant sections of the manuscript to improve transparency and accessibility, which was highlighted in RED FONT (Page 27).

16. The manuscript would benefit from a dedicated discussion of its limitations.

**Response**: Thank you very much for this important suggestion. We have appended TWO paragraphs to the discussion, which are highlighted in RED FONT (Page 24).

17. There are numerous figures and tables throughout the Results section. Many provide limited additional insight beyond the accompanying quantitative evaluation metrics written in the text. Consider moving some of these figures to the supplementary material to improve readability.

**Response**: Thank you for this valuable suggestion. We have carefully reviewed the figures and tables and retained them because they provide complementary information essential for interpreting and comparing the experimental results.

**Reviewer 2 comments:**

Reviewer #2: This paper compares different machine learning and ensemble approaches to detect suicidal ideation in Amharic social media posts within Facebook and Telegram. This work addresses an important gap in this area, which is mostly English-focused. The authors have built and released a first Amharic dataset on suicidal ideation, and benchmarked various ML models. It is very interesting and critical work; however, I believe it has certain issues that need addressing before publication.

**Major points:**

1) While the data originates from social media and is therefore in the public domain, it has been scraped, manually annotated, and has now been publicly released as a labeled dataset. The statement that consent "was not necessarily mandatory" is not sufficient justification on its own. Measures such as excluding direct quotes and identifying details are a reasonable step, but given how sensitive this topic is, this warrants a more considered and explicit consent/ethics process than is currently documented. At minimum, the authors should name the approving ethics body (if any), describe the de-identification protocol in more depth, and directly justify the decision to publicly redistribute this specific dataset.

**Response**: Thank you for this important comment. We agree that the ethical issues. We have revised and appended the manuscript, which is highlighted in RED FONT in (Pages 8, 9 and 27)

2) The Related Works section is not critical enough in the way it is written. While the concluding sentence tries to bring it all together, it largely lists the studies sequentially rather than providing some critical evaluation of them. A stronger review would ideally derive each gap from specific study's limitations, and connect it to the authors' own research question. A more thematic grouping (Ml vs DL vs multimodal etc.) would let the authors compare like with like and build the “low-resource language gap” argument better, rather than asserting it once at the end. The related work and methods sections currently read as disconnected pieces rather than the review motivating the method.

**Response**: Thank you for your valuable comment. We have revised and appended two paragraphs to the manuscript, which is highlighted in RED FONT on pages 6 and 7)

3) In Section 4, the narrative reports Bi-LSTM accuracy of 82.6% and F1 of 81.4%, but Table 5 lists Bi-LSTM accuracy as 81.4% and F1 as 80.8%. This needs reconciling before publication since it currently contradicts the paper's own primary results.

**Response**: Thank you for your valuable comment. Section 4 Results has been revised and all reported accuracies now exactly match Table 5. Appended and highlighted in RED FONT

4) Table 1 (character normalization), Table 2 (suicidal keyword list), and the prominent words quoted in the Discussion section all display as empty boxes/placeholders, likely because of the format of the PDF of submitted file. Since this is being reviewed for an English-language journal, this is a real barrier to review. At the moment, it is impossible to assess whether the normalization scheme in Table 1 is doing sensible linguistic work, whether the keyword list in Table 2 is appropriately broad or overly narrow, or whether the "prominent words" cited in the Discussion actually support the interpretive claims made about them. I would ask the authors to fix the font issue so the Amharic renders correctly, and since most readers and reviewers won't read/understand Amharic, it would be good to provide English translations alongside the Amharic terms in Tables 1–2 and in the word-cloud discussion, so it can let readers actually evaluate the linguistic contribution.

Response: Thank you for this important comment. We have **resolved the font/rendering issue and provided English translations alongside the Amharic terms** in Tables 2 and the relevant Discussion section. This ensures that the linguistic content and interpretations are accessible and evaluable to English-language readers. Appended Table 2, which was highlighted in Red FONT on (pages 10 -11)

5) The manuscript refers to "a domain expert," "an expert," and later "three separate topic experts". It is not clear whether these are the same people or different groups, and no qualifications are given for any of them. Are they mental health clinicians, linguists, social workers, or something else? "Domain expert" and "topic expert" should be explicitly defined and justified (such as clinical background, training in suicide risk assessment), since annotator expertise directly affects the validity of the annotations for a task like this.

**Response**: Thank you for this important comment. We have revised the manuscript to use the term **“domain expert” consistently throughout** and clarified their role and relevant qualifications. This change removes the previous ambiguity regarding “expert,” “topic expert,” and “domain expert” and improves the clarity and transparency of the annotation process

6) Confidence intervals should be added to all reported results. Several models are separated by less than a percentage point. Reporting CIs (or SD across the 10 folds) throughout Tables 5, 6, and 8 would let readers see whether these differences are statistically meaningful or overlapping noise.

**Response**: Thank you for this valuable suggestion. We acknowledge the importance of reporting variability measures. However, our study used **10-fold cross-validation with mean performance metrics**, and confidence intervals or standard deviations were not included in the original experimental design. We have clarified this limitation in the revised manuscript.

7) "Broad annotation standard that had been established" is unexplained. The manuscript references this standard without describing what it consists of, where or by whom it was established, or providing a citation. If it is based on an existing published annotation framework, that source should be cited. If it was developed by the authors themselves for this study, that should be stated explicitly along with its criteria, rather than referred to as if it were a known, pre-existing standard.

**Response:** Thank you for this helpful comment. We have clarified the **annotation standard and added the relevant citation** in the revised manuscript. We also briefly described the criteria and source of the standard to make its basis and application clear to readers, which was appended and highlighted in RED FONT (page 8)

8) Annotators used a list of 111 "suicidal indicator" words. If that same list also fed into feature engineering, the models may just be detecting keywords rather than real ideation, thereby inflating performance and weakening the claim of capturing "nuanced indicators." Authors should clarify this and test performance without keyword matches.

**Response:** Thank you for this important comment. We have clarified that the **111 suicidal indicator words were used only to support the annotation process and were not used as features or inputs for model training.** Therefore, the models did not rely on direct keyword matching, and the reported performance is based on the learned features rather than the indicator-word list.

9) There seems to be no description of how the keyword list was constructed. The paper doesn't explain how the 111 suicidal indicator terms were selected. were they literature-derived, clinician-generated, corpus-driven, or something else? This matters both for judging the annotation's validity and for assessing the circularity concern raised in the point above.

Response: Thank you for this valuable comment. We have clarified that the **111 suicidal indicator terms were literature-derived**, based on previously published studies and established terminology related to suicidal ideation.

10) Future work could consider incorporating emojis. Emojis are widely used on social media to convey emotional tone and intent, and could carry meaningful signal for suicidal ideation detection. The authors could state this as a limitation or future direction alongside their existing suggestions for multimodal expansion.

Response: Thank you for this valuable suggestion. We agree that emojis may provide meaningful emotional and contextual cues in social media content. We have **already incorporated the consideration of emojis as a limitation and future research direction** alongside the proposed multimodal extensions.

**Minor points:**

11) This sentence is repeated - "An expert used broad suicidal keywords.."
    - "The same study in [6]12]" - unclear citation. This formatting error makes it hard to tell which reference is actually being cited

Response: Thank you for pointing this out. We have **removed the repeated sentence and corrected the citation formatting and numbering** to ensure that the intended reference is clear and consistent throughout the manuscript.

12) Typo in the paragraph under Figure 10. The final sentence ends with "approache" instead of "approach" and needs correcting.

Response: Thank you for your comment. We have **corrected the typographical error from “approache” to “approach”**

13) Minor formatting - missing spaces between figure references. Multiple instances throughout the text run figure citations together without a space (e.g., "Figure5and Figure 6," "Figure7and Figure 8")

Response: Thank you for your valuable comment. We have **corrected the missing spaces between figure references throughout the manuscript** and ensured consistent formatting of all figure citations.

In general, we sincerely thank the Editor and Reviewers for their careful evaluation and highly constructive comments. We believe that the revised manuscript has been substantially strengthened through improved methodological clarity, corrected reporting, enhanced discussion of limitations, revised ethical and data availability statements, improved presentation, and corrected formatting. We hope that the revised version satisfactorily addresses all concerns and is now suitable for publication in Scientific Reports.
