# Supplementary material for: A comparative analysis of machine learning, deep learning, and ensemble learning models for suicidal ideation detection on social media
Source: PLOS Ment Health. 2026 Oct 22;3(10):e0000721. doi: 10.1371/journal.pmen.0000721 (PMC13690196; doi:10.1371/journal.pmen.0000721)
Supplement: Peer review history

# Peer review: Decision Letter 0

**DOI:** 10.1371/journal.pmen.0000721.r001

**Authors:** Ariel Soares Teles

23 Jul 2026

PMEN-D-26-00351

Comparing Machine and Deep Learning Approaches for Suicidal Ideation Detection in Amharic Social Media

PLOS Mental Health

Dear Dr. Wgiorgis,

Thank you for submitting your manuscript to PLOS Mental Health. After careful consideration, we feel that it has merit but does not fully meet PLOS Mental Health’s publication criteria as it currently stands. Therefore, we invite you to submit a revised version of the manuscript that addresses the points raised during the review process.

Consider all the comments provided by the reviewers.

Please submit your revised manuscript by Sep 21 2026 11:59PM. If you will need more time than this to complete your revisions, please reply to this message or contact the journal office at mentalhealth@plos.org. When you're ready to submit your revision, log on to https://www.editorialmanager.com/pmen/ and select the 'Submissions Needing Revision' folder to locate your manuscript file.

Please include the following items when submitting your revised manuscript:

- A letter that responds to each point raised by the editor and reviewer(s). You should upload this letter as a separate file labeled 'Response to Reviewers'.
- A marked-up copy of your manuscript that highlights changes made to the original version. You should upload this as a separate file labeled 'Revised Manuscript with Track Changes'.
- An unmarked version of your revised paper without tracked changes. You should upload this as a separate file labeled 'Manuscript'.

Guidelines for resubmitting your figure files are available below the reviewer comments at the end of this letter.

As the corresponding author, your ORCID iD is verified in the submission system and will appear in the published article. PLOS supports the use of ORCID, and we encourage all coauthors to register for an ORCID iD and use it as well. Please encourage your coauthors to verify their ORCID iD within the submission system before final acceptance, as unverified ORCID iDs will not appear in the published article. *Only*  the individual author can complete the verification step; PLOS staff *cannot*  verify ORCID iDs on behalf of authors.

We look forward to receiving your revised manuscript.

Kind regards,

Ariel Soares Teles

Academic Editor

PLOS Mental Health

Journal Requirements:

1. In your Methods section, please include additional information about your dataset and ensure that you have included a statement specifying whether the collection and analysis method complied with the terms and conditions for the source of the data.

2. Please note that PLOS Mental Health has specific guidelines on code sharing for submissions in which author-generated code underpins the findings in the manuscript. In these cases, we expect all author-generated code to be made available without restrictions upon publication of the work. Please review our guidelines at https://journals.plos.org/mentalhealth/s/materials-and-software-sharing#loc-sharing-code and ensure that your code is shared in a way that follows best practice and facilitates reproducibility and reuse.

3. Please provide separate figure files in .tif or .eps format.

For more information about figure files please see our guidelines:  LINK

https://journals.plos.org/digitalhealth/s/figures

https://journals.plos.org/mentalhealth/s/figures

https://journals.plos.org/mentalhealth/s/figures#loc-file-requirements

4. Please provide a complete Data Availability Statement in the submission form, ensuring you include all necessary access information or a reason for why you are unable to make your data freely accessible. If your research concerns only data provided within your submission, please write "All data are in the manuscript and/or supporting information files" as your Data Availability Statement.

If the reviewer comments include a recommendation to cite specific previously published works, please review and evaluate these publications to determine whether they are relevant and should be cited. There is no requirement to cite these works unless the editor has indicated otherwise.

Additional Editor Comments (if provided):

[Note: HTML markup is below. Please do not edit.]

Reviewers' comments:

Reviewer's Responses to Questions

**Comments to the Author**

1. Does this manuscript meet PLOS Mental Health’s publication criteria?

Reviewer #1: Partly

Reviewer #2: Yes

**********

2. Has the statistical analysis been performed appropriately and rigorously?

Reviewer #1: I don't know

Reviewer #2: Yes

**********

3. Have the authors made all data underlying the findings in their manuscript fully available (please refer to the Data Availability Statement at the start of the manuscript PDF file)?

The PLOS Data policy

Reviewer #1: Yes

Reviewer #2: Yes

**********

4. Is the manuscript presented in an intelligible fashion and written in standard English?

Reviewer #1: Yes

Reviewer #2: Yes

**********

Reviewer #1: Dear authors,

The manuscript explores an important and timely topic and offers a potentially valuable contribution through the development of a deep learning model for suicidal ideation detection in a low-resource language. The creation and evaluation of an Amharic-language dataset is a noteworthy contribution, as relatively little research has focused on suicidal ideation detection beyond English-language social media data. However, the manuscript contains several issues relating to its presentation, methodology, and overall framing that should be addressed before it is suitable for publication. I believe the following comments will improve the clarity, methodological transparency, and overall quality of the manuscript.

1. The manuscript would benefit from careful language editing to improve grammar, sentence structure, and readability. There are several instances where awkward phrasing affects clarity. Some examples are provided below.

In the Abstract "From the experimental results, the LSTM model outperformed and optimally the other models" should be revised to: "Based on the experimental results, the LSTM model performed optimally, outperforming the other models." In the introduction "According to the WHO, more than 800,000 persons die by suicide each year worldwide, which is equivalent to one death every 40 seconds."

Consider revising to: "According to the World Health Organization, more than 800,000 people worldwide die by suicide each year, equivalent to one death every 40 seconds."

2. The introduction provides useful background on suicide and social media; however, it does not adequately establish the specific research gap that motivates this study. The research questions appear rather abruptly at the end of the introduction before the reader has developed a clear understanding of the existing literature and its limitations. Although the manuscript states that research is limited in Ethiopia, this alone is not a sufficiently compelling justification. The introduction would be strengthened by explaining why existing models developed for high-resource languages are insufficient; the linguistic and cultural challenges associated with Amharic; why these challenges justify the development of a language-specific model. I also suggest integrating the current "Related Works" section into the introduction to produce a single, coherent narrative that culminates naturally in the research questions.

3. While the development of an Amharic-language suicidal ideation dataset appears to be one of the manuscript's primary contributions, this is not explicitly articulated. The manuscript would benefit from a concise statement explaining exactly how this work advances the existing literature beyond previous studies, rather than leaving the novelty for the reader to infer.

4. Please carefully review the reference list to remove duplicate entries and ensure that only the final published versions are cited. 12 and 13 appear to be the same paper, as does 7 and 22.

5. Throughout the Related Works section, studies are primarily introduced using phrases such as "A study in [citation]". It would improve readability to refer to studies using the first author's surname (e.g., "Basyouni et al."). More importantly, the section is largely descriptive rather than analytical. While previous studies are summarised, there is little critical evaluation of the literature or discussion of how the existing evidence informs and motivates the present study. The authors should synthesise the literature by identifying common trends, methodological limitations, and unresolved research gaps.

6. The stated research questions are not fully aligned with the methodology employed. In particular, RQ1 and RQ3 are review-oriented questions that would be more appropriate for a systematic or narrative review. However, the manuscript presents an experimental machine learning study. The research questions should instead focus on evaluating the proposed models and assessing their performance on the Amharic dataset

7. Please clarify what is meant by the phrase "methodical approach" in the opening sentence of Section 3 (Materials and Methods). The terminology is vague and should be explained or replaced with more precise language.

8. The manuscript occasionally refers to itself as conducting a "meta-analysis", which appears inaccurate. For example: "After identifying the problem, a review of the literature was conducted..." While the literature search appears systematic, the manuscript does not present the defining characteristics of a systematic review or meta-analysis (e.g., predefined eligibility criteria, PRISMA methodology, quality assessment, or quantitative synthesis). Instead, this section appears to present a narrative synthesis of previous work. Please clarify the purpose of the literature review and avoid referring to it as a meta-analysis unless a formal meta-analytic methodology has been undertaken.

9. The manuscript provides a detailed description of data cleaning and annotation but gives relatively little information regarding data collection. Please clarify or explain why the following can not be specified: the period during which Facebook and Telegram posts were collected; the specific groups or pages used (where appropriate); the scraping procedure; the inclusion and exclusion criteria used during data collection.

10. Please clarify how suicidal and non-suicidal posts were operationally defined during annotation. Although inter-rater agreement is reported, readers would benefit from additional information regarding: annotation guidelines; decision criteria; examples of annotated posts; and how semantic context was interpreted during the annotation process.

11. Was there a reason why more recent transformer-based multilingual baselines (e.g., multilingual BERT or XLM-RoBERTa) were not included? Given their widespread use in multilingual NLP, it would be useful to justify their omission or discuss this as a limitation.

12. Parts of the Results section contain material that would be more appropriate within the Introduction or Discussion. For example, the discussion comparing recent literature on English-language datasets and the evolution from conventional machine learning models to transformer-based models represents background literature rather than experimental findings. Consider relocating this material to improve the overall structure of the manuscript.

13. The Discussion currently reads largely as a repetition of the Results section. The authors could expand the discussion by: discussing the practical significance of the findings and explaining how the proposed framework advances current knowledge. In particular, the intended application of the model should be clarified. Is it intended for early identification of individuals at risk; population-level surveillance; clinical decision support; or social media monitoring. Discussing the practical applications, alongside the ethical implications of deploying such models, would considerably strengthen the manuscript.

14. Given that many of the reported performance differences are relatively small, these claims of one model "outperformed", "performed optimally", or was "superior" to the others should either be supported with appropriate statistical analyses or expressed more cautiously.

15. Although the manuscript states that publicly available social media data were used, the ethical considerations could be strengthened. As a reader I was curious about whether additional safeguards were implemented before publicly releasing the dataset. Given the sensitive nature of suicide-related data, further discussion is warranted

16. The manuscript would benefit from a dedicated discussion of its limitations

17. There are numerous figures and table throughout the Results section. Many provide limited additional insight beyond the accompanying quantitative evaluation metrics written in the text. Consider moving some of these figures to the supplementary material to improve readability.

Reviewer #2: This paper compares different machine learning and ensemble approaches to detect suicidal ideation in Amharic social media posts within Facebook and Telegram. This work addresses an important gap in this area, which is mostly English-focused. The authors have built and released a first Amharic dataset on suicidal ideation, and benchmarked various ML models. It is very interesting and critical work, however I believe it has certain issues that need addressing before publication.

Major points:

- While the data originates from social media and is therefore in the public domain, it has been scraped, manually annotated, and has now been publicly released as a labeled dataset. The statement that consent "was not necessarily mandatory" is not sufficient justification on its own. Measures such as excluding direct quotes and identifying details are a reasonable step, but given how sensitive this topic is, this warrants a more considered and explicit consent/ethics process than is currently documented. At minimum, the authors should name the approving ethics body (if any), describe the de-identification protocol in more depth, and directly justify the decision to publicly redistribute this specific dataset.

- The Related Works section is not critical enough in the way it is written. While the concluding sentence tries to bring it all together, it largely lists the studies sequentially rather than providing some critical evaluation of them. A stronger review would ideally derive each gap from specific study's limitations, and connect it to the authors' own research question. A more thematic grouping (Ml vs DL vs multimodal etc.) would let the authors compare like with like and build the “low-resource language gap” argument better, rather than asserting it once at the end. The related work and methods sections currently read as disconnected pieces rather than the review motivating the method.

- In Section 4, the narrative reports Bi-LSTM accuracy of 82.6% and F1 of 81.4%, but Table 5 lists Bi-LSTM accuracy as 81.4% and F1 as 80.8%. This needs reconciling before publication since it currently contradicts the paper's own primary results.

- Table 1 (character normalization), Table 2 (suicidal keyword list), and the prominent words quoted in the Discussion section all display as empty boxes/placeholders, likely because of the format of the PDF of submitted file. Since this is being reviewed for an English-language journal, this is a real barrier to review. At the moment, it is impossible to assess whether the normalization scheme in Table 1 is doing sensible linguistic work, whether the keyword list in Table 2 is appropriately broad or overly narrow, or whether the "prominent words" cited in the Discussion actually support the interpretive claims made about them. I would ask the authors to fix the font issue so the Amharic renders correctly, and since most readers and reviewers won't read/understand Amharic, it would be good to provide English translations alongside the Amharic terms in Tables 1–2 and in the word-cloud discussion, so it can let readers actually evaluate the linguistic contribution.

- The manuscript refers to "a domain expert," "an expert," and later "three separate topic experts". It is not clear whether these are the same people or different groups, and no qualifications are given for any of them. Are they mental health clinicians, linguists, social workers, or something else? "Domain expert" and "topic expert" should be explicitly defined and justified (such as clinical background, training in suicide risk assessment), since annotator expertise directly affects the validity of the annotations for a task like this.

- Confidence intervals should be added to all reported results. Several models are separated by less than a percentage point. Reporting CIs (or SD across the 10 folds) throughout Tables 5, 6, and 8 would let readers see whether these differences are statistically meaningful or overlapping noise.

- "Broad annotation standard that had been established" is unexplained. The manuscript references this standard without describing what it consists of, where or by whom it was established, or providing a citation. If it is based on an existing published annotation framework, that source should be cited. If it was developed by the authors themselves for this study, that should be stated explicitly along with its criteria, rather than referred to as if it were a known, pre-existing standard.

- Annotators used a list of 111 "suicidal indicator" words. If that same list also fed into feature engineering, the models may just be detecting keywords rather than real ideation, thereby inflating performance and weakening the claim of capturing "nuanced indicators." Authors should clarify this and test performance without keyword matches.

- There seems to be no description of how the keyword list was constructed. The paper doesn't explain how the 111 suicidal indicator terms were selected. were they literature-derived, clinician-generated, corpus-driven, or something else? This matters both for judging the annotation's validity and for assessing the circularity concern raised in the point above.

- Future work could consider incorporating emojis. Emojis are widely used on social media to convey emotional tone and intent, and could carry meaningful signal for suicidal ideation detection. The authors could state this as a limitation or future direction alongside their existing suggestions for multimodal expansion.

Minor points:

- this sentence is repeated - "An expert used broad suicidal keywords.."

- "The same study in [6]12]" - unclear citation. This formatting error makes it hard to tell which reference is actually being cited

- Typo in the paragraph under Figure 10. The final sentence ends with "approache" instead of "approach" and needs correcting

- Minor formatting - missing spaces between figure references. Multiple instances throughout the text run figure citations together without a space (e.g., "Figure5and Figure 6," "Figure7and Figure 8")

**********

what does this mean?). If published, this will include your full peer review and any attached files.

**Do you want your identity to be public for this peer review?**  If you choose “no”, your identity will remain anonymous but your review may still be made public.

For information about this choice, including consent withdrawal, please see our Privacy Policy

Reviewer #1: No

Reviewer #2: No

**********

[NOTE: If reviewer comments were submitted as an attachment file, they will be attached to this email and accessible via the submission site. Please log into your account, locate the manuscript record, and check for the action link "View Attachments". If this link does not appear, there are no attachment files.]

# Author comment: Author response to Decision Letter 1

**DOI:** 10.1371/journal.pmen.0000721.r002

23 Aug 2026

Attachment Submitted filename: Response File Reviewers Comments.docx

# Peer review: Decision Letter 1

**DOI:** 10.1371/journal.pmen.0000721.r003

**Authors:** Ariel Soares Teles

16 Sep 2026

PMEN-D-26-00351R1

A Comparative Analysis of Machine Learning, Deep Learning, and Ensemble Learning Models for Suicidal Ideation Detection on Social Media

PLOS Mental Health

Dear Dr. Wgiorgis,

Thank you for submitting your manuscript to PLOS Mental Health. After careful consideration, we feel that it has merit but does not fully meet PLOS Mental Health’s publication criteria as it currently stands. Therefore, we invite you to submit a revised version of the manuscript that addresses the points raised during the review process.

Please, check the characters in tables 1 and 2.

Please submit your revised manuscript by Oct 16 2026 11:59PM. If you will need more time than this to complete your revisions, please reply to this message or contact the journal office at mentalhealth@plos.org. When you're ready to submit your revision, log on to https://www.editorialmanager.com/pmen/ and select the 'Submissions Needing Revision' folder to locate your manuscript file.

Please include the following items when submitting your revised manuscript:

- A letter that responds to each point raised by the editor and reviewer(s). You should upload this letter as a separate file labeled 'Response to Reviewers'.
- A marked-up copy of your manuscript that highlights changes made to the original version. You should upload this as a separate file labeled 'Revised Manuscript with Track Changes'.
- An unmarked version of your revised paper without tracked changes. You should upload this as a separate file labeled 'Manuscript'.

Guidelines for resubmitting your figure files are available below the reviewer comments at the end of this letter.

As the corresponding author, your ORCID iD is verified in the submission system and will appear in the published article. PLOS supports the use of ORCID, and we encourage all coauthors to register for an ORCID iD and use it as well. Please encourage your coauthors to verify their ORCID iD within the submission system before final acceptance, as unverified ORCID iDs will not appear in the published article. *Only*  the individual author can complete the verification step; PLOS staff *cannot*  verify ORCID iDs on behalf of authors.

We look forward to receiving your revised manuscript.

Kind regards,

Ariel Soares Teles

Academic Editor

PLOS Mental Health

Journal Requirements:

If the reviewer comments include a recommendation to cite specific previously published works, please review and evaluate these publications to determine whether they are relevant and should be cited. There is no requirement to cite these works unless the editor has indicated otherwise.

Please review your reference list to ensure that it is complete and correct. If you have cited papers that have been retracted, please include the rationale for doing so in the manuscript text, or remove these references and replace them with relevant current references. Any changes to the reference list should be mentioned in the rebuttal letter that accompanies your revised manuscript. If you need to cite a retracted article, indicate the article’s retracted status in the References list and also include a citation and full reference for the retraction notice.

Additional Editor Comments (if provided):

[Note: HTML markup is below. Please do not edit.]

Reviewers' comments:

Reviewer's Responses to Questions

**Comments to the Author**

Reviewer #1: All comments have been addressed

Reviewer #2: All comments have been addressed

**********

publication criteria?

Reviewer #1: Yes

Reviewer #2: Yes

**********

3. Has the statistical analysis been performed appropriately and rigorously?

Reviewer #1: Yes

Reviewer #2: Yes

**********

4. Have the authors made all data underlying the findings in their manuscript fully available (please refer to the Data Availability Statement at the start of the manuscript PDF file)?

The PLOS Data policy

Reviewer #1: Yes

Reviewer #2: Yes

**********

5. Is the manuscript presented in an intelligible fashion and written in standard English?

Reviewer #1: Yes

Reviewer #2: Yes

**********

**Review Comments to the Author**

Reviewer #1: This has been a nice piece of work to review. Thank you for the opportunity to review it. All of the comments I made have been resolved in a very thoughtful way. The only thing that I think needs to be resolved is that the Amharic characters in table 1 and 2 are not visible. They appear in both uploaded files as boxes. My suggestion would be to use a figure/screenshot of the table. But this might be something to bring up with the editor to see if they have any suggestions on how to solve this problem as I feel it is incredibly important that these characters can be seen.

Reviewer #2: The authors have addressed all my comments, and I am now satisfied with the revisions.

**********

what does this mean?). If published, this will include your full peer review and any attached files.

**Do you want your identity to be public for this peer review?**  If you choose “no”, your identity will remain anonymous but your review may still be made public.

For information about this choice, including consent withdrawal, please see our Privacy Policy

Reviewer #1: No

Reviewer #2: No

**********

[NOTE: If reviewer comments were submitted as an attachment file, they will be attached to this email and accessible via the submission site. Please log into your account, locate the manuscript record, and check for the action link "View Attachments". If this link does not appear, there are no attachment files.]

# Author comment: Author response to Decision Letter 2

**DOI:** 10.1371/journal.pmen.0000721.r004

18 Sep 2026

Attachment Submitted filename: 3Response File to.pdf

# Editor report: Decision Letter 2

**DOI:** 10.1371/journal.pmen.0000721.r005

**Authors:** Ariel Soares Teles

22 Sep 2026

A Comparative Analysis of Machine Learning, Deep Learning, and Ensemble Learning Models for Suicidal Ideation Detection on Social Media

PMEN-D-26-00351R2

Dear Dr Wgiorgis,

We are pleased to inform you that your manuscript 'A Comparative Analysis of Machine Learning, Deep Learning, and Ensemble Learning Models for Suicidal Ideation Detection on Social Media' has been provisionally accepted for publication in PLOS Mental Health.

Before your manuscript can be formally accepted you will need to complete some formatting changes, which you will receive in a follow up email. A member of our team will be in touch with a set of requests.

Please note that your manuscript will not be scheduled for publication until you have made the required changes, so a swift response is appreciated.

IMPORTANT: The editorial review process is now complete. PLOS will only permit corrections to spelling, formatting or significant scientific errors from this point onwards. Requests for major changes, or any which affect the scientific understanding of your work, will cause delays to the publication date of your manuscript.

If your institution or institutions have a press office, please notify them about your upcoming paper to help maximize its impact. If they'll be preparing press materials, please inform our press team as soon as possible -- no later than 48 hours after receiving the formal acceptance. Your manuscript will remain under strict press embargo until 2 pm Eastern Time on the date of publication. For more information, please contact mentalhealth@plos.org.

Thank you again for supporting Open Access publishing; we are looking forward to publishing your work in PLOS Mental Health.

Best regards,

Ariel Soares Teles

Academic Editor

PLOS Mental Health

***********************************************************

Reviewer Comments (if any, and for reference):
